# Spatiotemporal variation in cell proliferation patterns during arthropod axial elongation

**DOI:** 10.1038/s41598-020-79373-0

**Published:** 2021-01-11

**Authors:** Rodrigo E. Cepeda, John B. Terraza, Renato V. Pardo, Valentina Núñez-Pascual, Marco Mundaca-Escobar, Andres F. Sarrazin

**Affiliations:** Instituto de Química, Pontificia Universidad Católica de Valparaíso, Valparaíso, Chile

**Keywords:** Developmental biology, Pattern formation

## Abstract

An elongated and segmented body plan is a common morphological characteristic of all arthropods and is probably responsible for their high adaptation ability to diverse environments. Most arthropods form their bodies by progressively adding segments, resembling vertebrate somitogenesis. This sequential segmentation relies on a molecular clock that operates in the posterior region of the elongating embryo that combines dynamically with cellular behaviors and tissue rearrangements, allowing the extension of the developing body along its main embryonic axis. Even though the molecular mechanisms involved in elongation and segment formation have been found to be conserved in a considerable degree, cellular processes such as cell division are quite variable between different arthropods. In this study, we show that cell proliferation in the beetle *Tribolium castaneum* has a nonuniform spatiotemporal patterning during axial elongation. We found that dividing cells are preferentially oriented along the anterior–posterior axis, more abundant and posteriorly localized during thoracic segments formation and that this cell proliferation peak was triggered at the onset of axis elongation. This raise in cell divisions, in turn, was correlated with an increase in the elongation rate, but not with changes in cell density. When DNA synthesis was inhibited over this period, both the area and length of thoracic segments were significantly reduced but not of the first abdominal segment. We discuss the variable participation that different cell division patterns and cell movements may have on arthropod posterior growth and their evolutionary contribution.

## Introduction

The development of a typical feature of all arthropods, their segmented body, is achieved by a highly coordinated combination of genetic programming and dynamic cellular behaviors. In the vinegar fly *Drosophila melanogaster*, almost all body segments are patterned by the practically simultaneous subdivision of the syncytial blastoderm before germband extension^1,2^ whereas in the vast majority of arthropods, most segments arise sequentially in an anterior to posterior progression from an apparently undifferentiated region located at the caudal end of the embryo/larva (known as the segment addition zone—SAZ—or growth zone) at the same time that the body’s main axis is lengthening^3,4^.

It is already known that* Drosophila* axial elongation relies mostly on cell rearrangements or oriented cell divisions, depending on the region along the germband and the temporal phase of elongation analyzed^5–7^. Among sequentially segmenting arthropods, the extent of participation of cell proliferation and cell intercalations is less known. Few studies have addressed these issues, and they have shown that the proportional contribution of each cellular process to elongation seems to vary substantially from taxon to taxon, which could explain—at least in part—their high morphological diversity^8^.

In the centipede *Strigamia maritima*, the large number of cells contained in the terminal disc from which the trunk segments are formed suggests that cell divisions are dispensable and that convergent extension might be the driving force of axial elongation in myriapods^9,10^. In the common house spider *Parasteatoda tepidariorum*, germband elongation was described by Hemmi and colleagues as a combination of cell movements and cell proliferation, conferring higher contribution of cell movements to the formation of the presumptive opisthosomal region (posterior part of the body in chelicerates), without recognizable patterns of cell division in this posterior region^11^. On the other hand, in Malacostraca, the largest of the six classes of the paraphyletic group of crustaceans^12–14^, cell proliferation in the post-naupliar germband (region posterior to the mandibular segment) shows a highly conserved stereotyped pattern where few cells form the complete set of trunk segments by ordered divisions, making clear the essential role of cell proliferation in the majority of crustaceans^15–17^. In addition, recent findings in a branchiopod crustacean more closely related to insects than malacostracans^18^, the fairy shrimp *Thamnocephalus platyurus*, revealed that posteriorly localized cell proliferation is not high but shows three stable domains along the anterior–posterior axis. While the most posterior region of the SAZ displays reduced numbers of uncoordinated dividing cells and the anterior region of the SAZ exhibits almost no proliferation, the most recently formed segment shows higher levels of cycling cells undergoing S-phase in synchrony. Moreover, the authors observed that these distinct cell cycle domains matched well with the expression patterns of *caudal* and *wnt* genes^19^.

Based on similar findings in a sequentially segmenting insect, the hemipteran *Oncopeltus fasciatus*, the same collaborative team proposed a model that correlated gene expression domains within the SAZ with different cell proliferation levels. They related the low level of cell division in the anterior part of the SAZ with the dynamic expression of pair-rule and Notch signaling genes, while the domains of relatively high levels of proliferation flanking it at both anteriorly and posteriorly were correlated with the expression of segment polarity genes and the stable expression of *caudal* and *even-skipped* genes, respectively^20^. In previous work from our group, we found that in the beetle *Tribolium castaneum*, there seems to be no apparent coordination between the generation of the segmentation pattern and cell proliferation since the inhibition of DNA synthesis by aphidicolin and hydroxyurea produced shorter embryos without disrupting segment formation^21^. Therefore, despite the considerable degree of conservation of the molecular mechanisms underlying both segmentation and elongation within insects and even arthropods^22^, patterns of cellular processes such as cell proliferation seem to be less conserved or at least show a high degree of variability.

In this study, we examined in detail the contribution of cell proliferation to axial elongation. We first describe that in the *Tribolium* germband, cell division has nonuniform spatiotemporal patterning during much of axial elongation. In contrast to the observations in *Oncopeltus*, where cell divisions in the posterior region are spatially patterned—and temporally stable—throughout elongation^20^, we discovered a noteworthy peak of dividing cells at the time of thoracic segments specification that was most likely triggered at the beginning of elongation. We also found that this peak coincided with an increase in the rate of germband lengthening as well as with the proper formation of the thoracic segments. Consistent the former observation, Nakamoto et al*.*^23^ observed that the periodicity of segment addition in the *Tribolium* germband was not constant, showing different rates of *engrailed* stripe appearance during elongation. Interestingly, whereas they detected acceleration of segmentation at the transition from thoracic to abdominal segments, we found an increase in the rate of elongation during thoracic segments formation. Although there is enough evidence that segmental patterning is coupled to germband extension—most likely through cell rearrangements—^23–25^, our results suggests that cell division-driven elongation and segmentation probably respond to different regulatory inputs in *Tribolium*. Furthermore, based on the idea that the contribution of cell proliferation to axial elongation could be concealed by cell size reduction or increased by cell divisions oriented along the anterior–posterior axis, we measured the possible changes in cell compaction and preferential orientation of cell divisions. While there was no evidence for changes in cell density during elongation, we found a significantly higher proportion of cell divisions oriented along the anterior–posterior axis of the extending embryo.

The findings of the present study, together with previous evidence, demonstrate that during axial elongation, patterns of cell division are highly variable among different arthropods as well as quite conserved between individuals of the same species. We surmise that measured changes in the spatiotemporal regulation of cell proliferation during evolution, through shifts in the relative position (heterotopy) or timing (heterochrony) of this cellular process during development, could account for some of the dramatic morphological differences we find among arthropods without the need of large changes at the genetic level.

## Results

### Nonuniform spatiotemporal patterning of cell division during elongation

**Figure 1:**
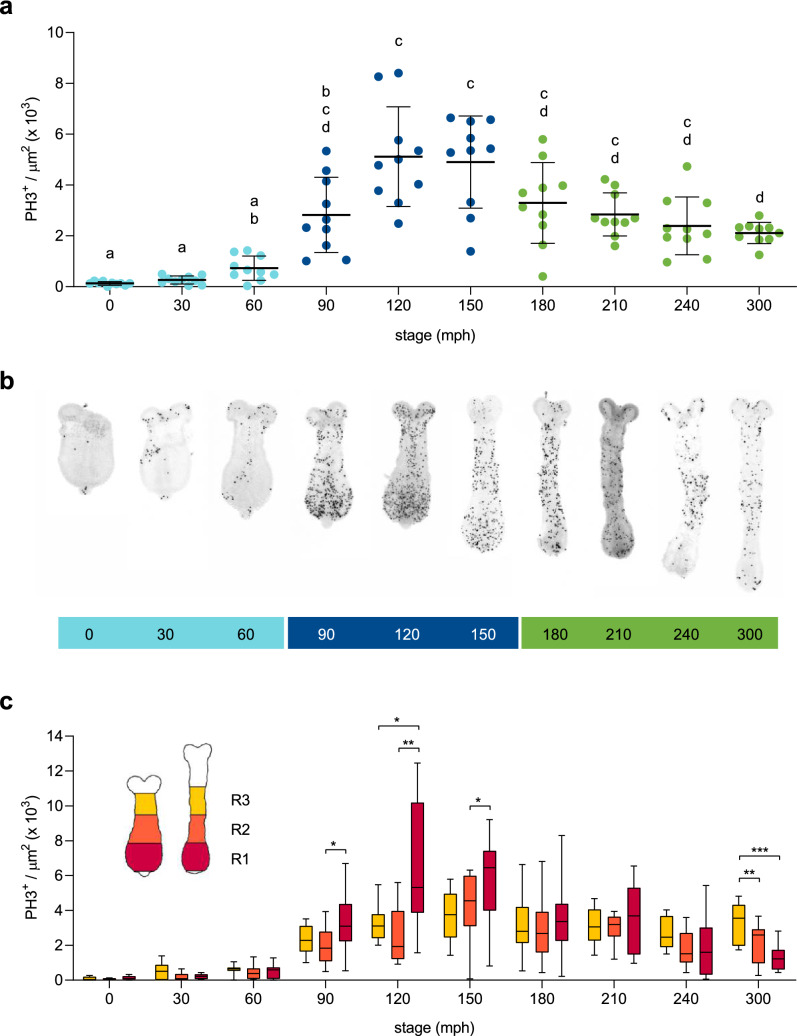
Nonuniform spatiotemporal patterning of cell division during elongation. (**a**) Three temporal phases of different levels of proliferation were found after PH3^+^ cells quantification (normalized by area × 10^3^) every 30 min from 0 to 300 mph (n = 10 at each stage; 270 mph was not counted). Minimum levels (turquoise) precede a peak of dividing cells (blue) that is followed by intermediate amounts of PH3^+^ cells (green). (**b**) Representative pictures of PH3 stained embryos at each stage analyzed, ordered from 0 to 300 mph (red fluorescent images were converted to black and white and color-inverted to improve visualization). (**c**) The spatial patterning of cell proliferation at each stage was obtained after PH3^+^ cells quantification at three consecutive regions along the elongating embryo (R1–R2–R3 from posterior to anterior; see the schematic diagram). All embryos showed are dorsally oriented. Anterior is to the top. Error bars indicate standard deviation (SD) of the mean (n = 10). Different letters in (**a**) represent groups with statistically significant differences according to a Brown-Forsythe and Welch ANOVA test (*p* < 0.05). Statistical analysis in (**c**) was performed between each region at every stage. Asterisks indicate statistically significant differences according to a two-way ANOVA with post hoc Tukey's multiple comparisons test; *p < 0.05, **p < 0.01 and ***p < 0.001. Corresponding *p*-values are showed in Supplementary Table S2.

Given that our previous analysis showed considerable contribution of cell proliferation to *Tribolium* germband elongation^21^, we wondered whether the amount of cell divisions would be regular in time and space. For this purpose we decided to map the spatiotemporal pattern of cell divisions during much of *Tribolium* axial elongation, starting at the formation of the first *Tc-engrailed* (*Tc-en*) stripe or mandibular segment (0 min post-horseshoe stage; 0 mph) and finishing at 300 mph, approximately when the 9th *Tc-en* stripe (3^rd^ abdominal segment) is already formed. We performed immunostaining against the mitotic marker phospho-histone H3 (PH3) in order to quantify in more detail (every 30 min) the total number of proliferating cells (normalized by area/volume) along the elongating embryo (Fig. 1, Supplementary Table S1; n = 10 for each time point analyzed). Our measurements shed light on the temporal and spatial cell division pattern that emerge during germband extension.

Temporally, three phases of different levels of proliferation were found (Fig. 1a,b). At an early stage (0–60 mph; first phase) we found that very few cells were labelled (5.3% of the total PH3^+^ cells recorded). Then, after a sudden leap in the number of PH3 labelled cells from 60 to 90 mph (3.4-fold higher, from 42 ± 28 to 144 ± 78 PH3^+^ cells in average), we found a peak of cells in mitosis between 120 and 150 mph (average: 254 ± 101 and 271 ± 91 PH3^+^ cells, respectively), around the formation of the fifth *Tc-en* stripe (2nd thoracic segment). This peak represents 41.1% of the total PH3^+^ cells recorded (5249 out of 12,787 cells from 100 embryos analyzed) that entered cell division during the whole period analyzed (5 h covering the formation of one to 9 *Tc-en* stripes). For statistical analysis, we normalized to embryo size by dividing the total number of PH3^+^ cells by each germband area ((PH3^+^ cells/µm^2^) × 10^3^; see “Methods” for details and Supplementary Table S1). Using these values, we found a second phase of proliferation, with significant differences between the peak of proliferating cells at 120–150 mph and the amount of PH3^+^ cells at 60 mph (*p* = 0.0019 for 120 mph and *p* = 0.0014 for 150 mph; Fig. 1a). Subsequently (third phase), we found that cell proliferation is maintained from 180 to 300 mph at an intermediate level with a gradual reduction as the embryo elongates until the last stage quantified (Supplementary Table S1), but without returning to the levels of the initial phase (statistical difference is maintained between 60 mph and all succeeding stages; Fig. 1a).

Spatially, PH3^+^ cells were assessed by both visual examination and statistical analysis along the anterior–posterior (AP) axis (Fig. 1c). First thing that caught our attention was the nonuniform distribution of dividing cells (see Supplementary Figs. S1–S10 for the complete set of pictures). At all stages analyzed, there was no obvious coordination between left and right sides of the embryo. Between 0 and 120 mph, PH3^+^ cells tend to accumulate at specific regions within the extending germband: head lobes (not considered in the quantification), middle line (putative mesoderm), caudal end (putative mesodermal precursor cells)^26^ and the segment addition zone (SAZ) or growth zone. From 150 to 300 mph, proliferating cells do not display any specific pattern and dividing cell domains are randomly distributed. However, what is visually evident is the progressively decrease in the number of PH3^+^ cells as the elongation progresses (Supplementary Figs. S6–S10).

In order to provide statistical support to the posteriorly localized proliferating cells that we observed at specific stages during the elongation process, we quantified PH3^+^ cells into three consecutive regions that divided the germband from posterior to anterior along its axis (R1, R2 and R3 regions; see the schematic diagram showed in Fig. 1c). When we compared the number of dividing cells in each region at every stage analyzed (Supplementary Table S1), we found a general tendency of higher levels of labeled cells present at the most posterior region (R1) between 90 and 150 mph, approximately the same stages where the peak of proliferation was previously found (Fig. 1a,b). After area normalization, this tendency was statistically significant in each of these stages (Fig. 1c; R1 > R2 at 90 mph, *p* = 0.0204; R1 > R2 and R1 > R3 at 120 mph, *p* = 0.0038 and *p* = 0.0252, respectively; R1 > R2 at 150 mph, *p* = 0.0176). In addition, we discovered an opposite distribution at the last stage analyzed (R3 > R2 and R3 > R1 at 300 mph, *p* = 0.0013 and p = 0.0007, respectively), with more dividing cells located at a more anterior position (Fig. 1c).

### Cell proliferation peak is triggered at the beginning of axial elongation

**Figure 2:**
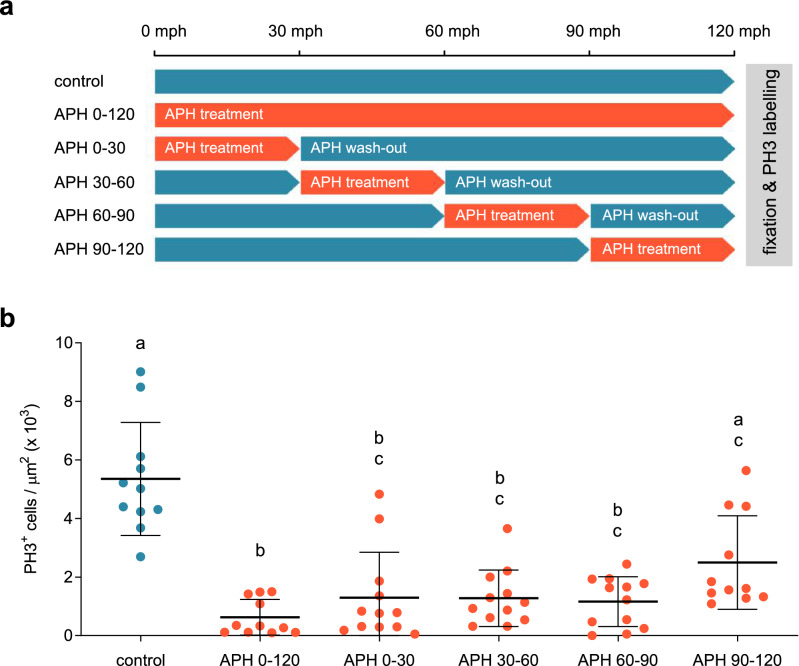
Cell proliferation peak is triggered at the beginning of axial elongation. (**a**) Experimental schematic for time window Aphidicolin treatments. Aphidicolin incubation windows and washouts are highlighted in orange and blue, respectively. All treatments lasted 2 h and were followed by embryo fixation and anti-PH3 antibody staining. (**b**) Number of PH3^+^ cells (normalized by area × 10^3^) measured at 120 mph after each treatment. Error bars indicate SD of the mean (n = 11–12). Different letters represent groups with statistically significant differences according to a Kruskal–Wallis test and Dunn’s multiple comparisons test (*p* < 0.05). Corresponding *p*-values are showed in Supplementary Table S2.

Since a large population of dividing cells are concentrated and posteriorly localized at a specific period during elongation (Fig. 1), we wondered about the time when cell cycle should be induced (or upregulated) in order to enter into replication and reach this cell proliferation peak. For that we performed whole embryo incubations at different time windows with the reversible DNA synthesis inhibitor Aphidicolin (APH)^21,27,28^, followed by PH3 labelling at 120 mph, the time at the beginning of the peak of high proliferation mentioned before (Fig. 2a; n = 11–12 for each time window essay). First, we compared the number of cell divisions at 120 mph between untreated control and 2-h treated germbands and we found a statistically significant decrease in PH3^+^ cells and germband length, as expected (Fig. 2b)^21^. The explanation to this effect is that most proliferating cells found at 120 mph are exposed to blockage by APH because they were passing through the border of G1 and S phases between 0 and 120 mph. However, when we incubated dissected germbands with APH for only 30 min time windows followed by washout with insect medium in order to eliminate the APH (Fig. 2a), we discovered that the effect of blocking G1/S-phase in three of these cases (0–30, 30–60 and 60–90 mph time windows) was very similar to what we obtained after 2-h treatment. Only APH incubation between 90 and 120 mph showed a partial inhibition of cell division (Fig. 2b). All these mean that between 0 and 90 mph the same cells are cycling through the G1/S-phase and only 30 min (90–120 mph time window) before PH3 measurement at 120 mph, some cells leave the blockage by probably entering the G2/M-phase. These results suggest that the triggering of DNA replication that become the peak of posteriorly localized cell divisions around 120–150 mph, takes place around 1–2 h before.

### Cell proliferation peak correlates with an increasing in the elongation rate and proper thoracic segments formation

**Figure 3:**
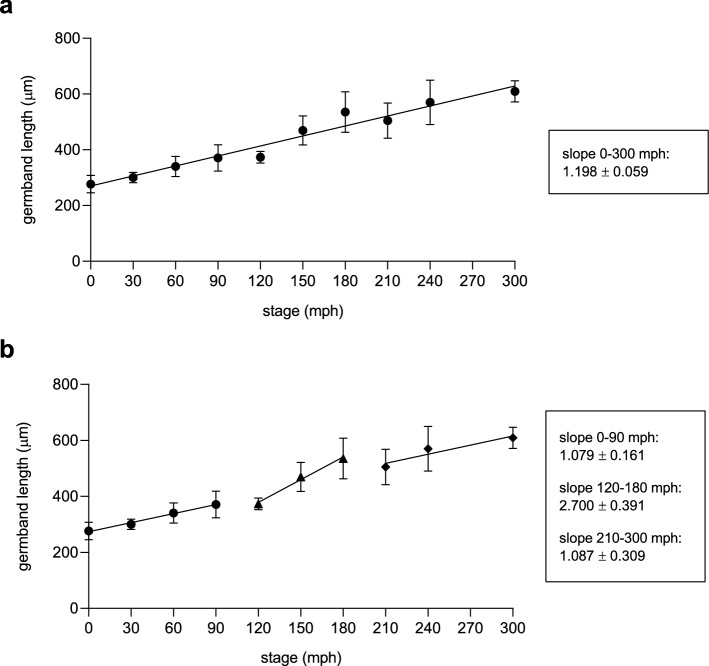
Cell proliferation peak correlates with an increasing in the elongation rate. (**a**) Germband’s length throughout elongation measured every 30 min from 0 to 300 mph (n = 10 at each stage; 270 mph was not counted) after linear regression showed an average rate of elongation of 1.198 ± 0.059 µm/min. The slope represents the rate of elongation at each period. (**b**) Linear regression applied to separated periods of elongation showed 2 different slopes. The period between 120–180 mph showed to be statistically different to 0–90 mph (*p* = 0.0001) and 210–300 mph (*p* = 0.0029). All data were fitted with a linear regression. Error bars indicate SEM (n = 10).

In view of the results obtained, we wondered whether there might be an effect on axial elongation and segmentation that could be attributed to the nonuniform distribution of proliferating cells. Therefore, we first measured the changes on germband’s length throughout elongation every 30 min starting at 0 mph (Fig. 3a). After all data were fitted with a linear regression, we found an average rate of elongation of 1.2 ± 0.06 µm/min. However, the analysis of changes in germband length revealed that axial elongation is far from gradual. During the period analyzed, where germbands at least double their length (from 276.7 ± 31.1 at 0 mph µm to 609.3 ± 37.98 µm at 300 mph in average), we found a sudden leap in the elongation rate between 120 and 150 mph (Supplementary Fig. S11). When we checked in more detail (Fig. 3b), we were able to identify a transition in slope at the 120–180 mph period (slope of 2.7), that is significantly different *(p* < 0.0018) with respect to the periods just before (0–90 mph; slope of 1.08) and after (210–300 mph; slope of 1.09), revealing a correlation between the time of the proliferation peak—in other words, the sudden raise in cells number that takes place between 120 and 150 mph—and the increase in the elongation rate showed here.

**Figure 4:**
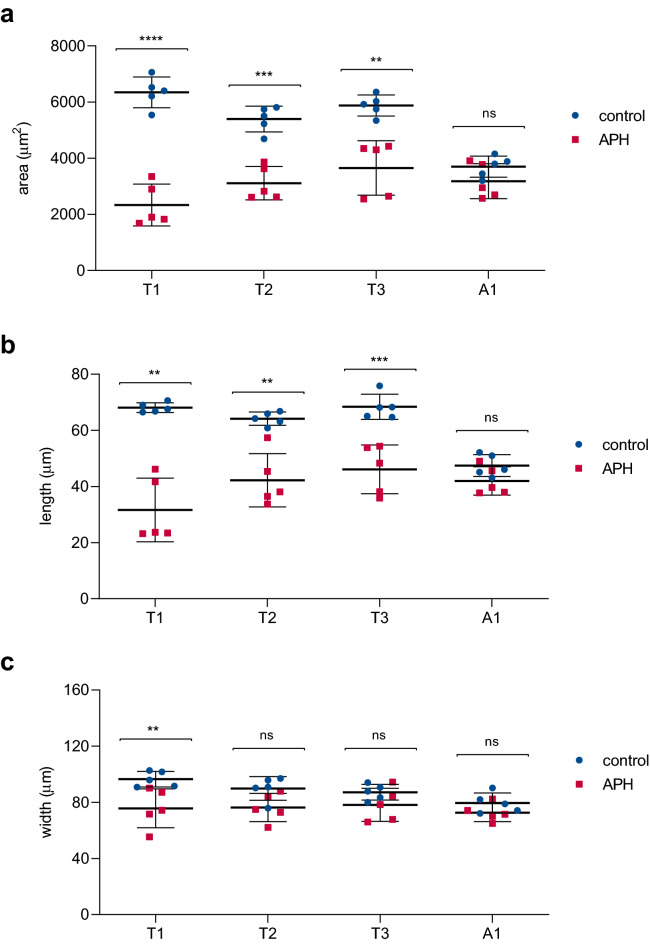
Cell proliferation peak is necessary for proper thoracic segments formation. Comparison of different parameters, area (**a**), length (**b**) and width (**c**), from the three thoracic (T1, T2 and T3) and the first abdominal (A1) segments between APH treated germbands (red squares) and controls (blue dots). Dissected germbands were exposed to the APH treatment or DMSO (controls) during 1-h followed by washout, another 4 h of incubation and finally fixation to subsequent *Tc-engrailed *in situ hybridization and analysis. Error bars represent SD of the mean (n = 5). Asterisks indicate statistically significant difference according to unpaired test t (**a**,**b**) and one-way ANOVA (**c**); *p < 0.05; **p < 0.01; ***p < 0.001 and ****p < 0.0001. Corresponding *p*-values are showed in Supplementary Table S2.

In addition, given that the largest increase in cell division covers the period of thoracic segments formation, we anticipated that these segments would be affected by the inhibition of DNA synthesis at just the time when the peak of proliferation is triggered (see Fig. 2). Thus, we incubated dissected embryos at 0 mph during 1 h with Aphidicolin followed by washout and subsequent incubation in insect medium for another 4 h until approximately the formation of the first abdominal segments. Consistently with our hypothesis, when we analyzed the size of T1, T2, T3 and A1 segments (thoracic segments 1, 2, 3 and abdominal segment 1, respectively) in control and treated embryos after *Tc-engrailed *in situ hybridization (Supplementary Fig. S12), we observed that the area and length of all thoracic segments were significantly reduced in APH-treated embryos (Fig. 4a,b), as well as their width, but to a lesser extent (Fig. 4c). However, when we compared the abdominal segment 1 between treated and control embryos, they appeared to be similar in area and length (Fig. 4).

### Preferred cell division orientation along the main axis during elongation without changes in cell compaction

Based on the idea that contribution to axial elongation by an increased number of cells could be minimized by cell compaction, we measured possible changes in cellular density along the elongating germband (Supplementary Fig. S13), considering the number of cellular nuclei that fit in a given area as a meaning of cellular compaction (see “Methods” for details). The statistical analysis obtained after comparing cellular densities at each stage quantified (from 0 to 210 mph), at three different positions along the germband, showed practically no significant changes during elongation.

**Figure 5:**
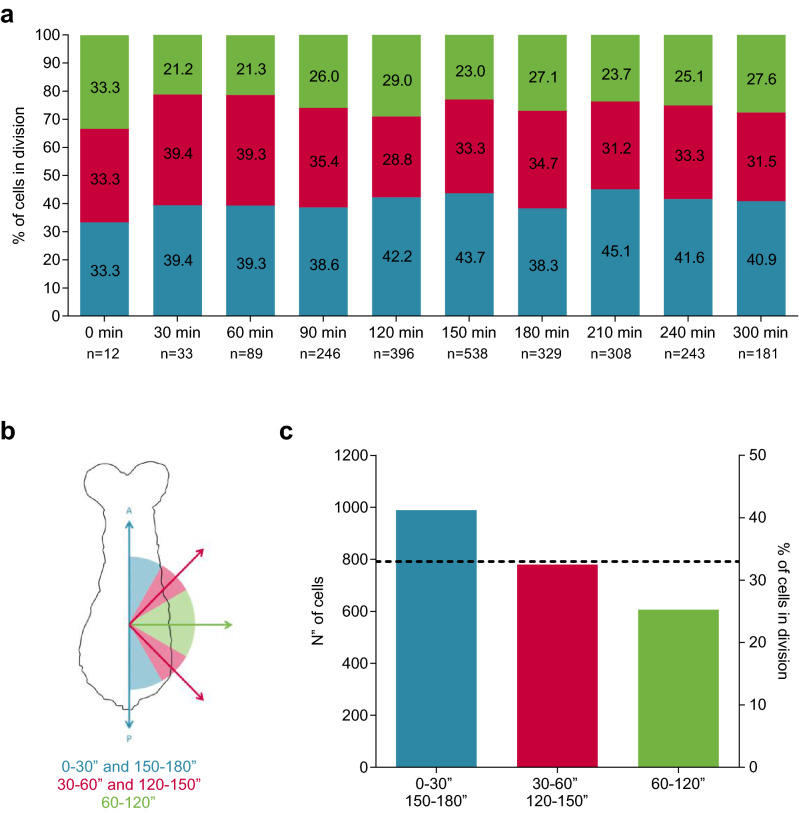
Cell division orientation has an anterior–posterior contribution to germband extension. (**a**) Percentage distribution of mitotic angles relative to the anterior–posterior axis (elongation axis) by stage and total distribution. Number of cells analyzed (n) is also shown. (**b**) Schematic diagram of a *Tribolium* germband showing the angle selection criteria and the color code. Blue: Mitotic angles between 0°–30° and 150°–180°; Red: 30°–60° and 120°–150°; Green: 60°–120°. (**c**) Total distribution analysis of cell division angles. The broken line represents the expected percentage if random cell divisions are present during elongation (~ 33%). The bars represent the amount of dividing cells that showed the corresponding angle range. Each group was analyzed by the Chi-square test of homogeneity, showing that the orientations were not distributed equally (*p* < 2.2 × 10^–16^).

Another cellular process that can be involved in germband elongation is oriented mitosis along the anterior–posterior axis^7,29^. In order to evaluate the possible preferential orientation of cellular divisions along embryo’s main axis, we measured the angle formed between the cell division axis and the anterior–posterior axis (see Supplementary Fig. S14) of all PH3^+^ cells that showed metaphase or anaphase figures at every stage analyzed (from 0 to 300 mph; approximately 20% of the total labelled cells). For the analysis (Fig. 5), we defined three angle ranges: Angles between 0°–30° and 150°–180° were considered parallel to the anterior–posterior axis; angles between 30°–60° and 120°–150° were considered oblique; angles between 60°–120° were considered perpendicular to the main axis (Fig. 5b). If the angles of cell division are random, each group of angles would be expected to be present in equivalent proportions with respect to the total of the measured orientations (33.3%, corresponding to 791.6 out of a total of 2375 cells). When analyzing the values of each of the three groups according to the Chi-square test of homogeneity (Fig. 5a), the results showed that the orientations were not distributed equally, with the group with orientation between 0°–30° and 150°–180° obtaining a higher proportion (41.6%, corresponding to 989 cells) and the 60°–120° group a smaller proportion (25.5%, corresponding to 606 cells) of dividing cells than expected (Fig. 5b). When ruling out the homogeneity of distribution of the orientations, it can be affirmed that there was a trend in the mitotic angles measured to be parallel to the anterior–posterior axis of the embryo. This tendency was maintained when the analysis was performed at different positions along the embryo at all stages analyzed (Supplementary Fig. S15), suggesting that a significant proportion of cell divisions oriented in the direction of germband growth are also contributing to embryo elongation.

## Discussion

Arthropod sequential segmentation has an intrinsically dynamic nature. It takes place at the same time that the posterior body elongates along its main axis, and therefore, the whole process results in a complex mix of cell behaviors and molecular mechanisms. When posterior elongation has been observed and analyzed by live imaging^30^, it has been evident that cell rearrangements and convergent cell movements play a central role in this process. At the same time, this and other studies of mitotic patterns during axial elongation and segmentation have revealed that cell division also plays a prominent role, but this contribution appears to be highly differential between species. Thus, it is becoming increasingly obvious that a spatially and temporally variable combination of both processes is clearly underlying arthropod posterior growth.

Few published studies in arthropods (we will exclude from the analysis the stereotyped cell division pattern of malacostracans) have addressed in detail the role that cell proliferation plays during sequential segmentation and axial elongation^19–21,23,31^. However, based on these works and direct/indirect evidence obtained from other arthropods^10,11,32–38^, we are already able to identify some shared characteristics. First, practically all arthropods studied show some degree of cell proliferation during elongation. Second, some of them present a spatially patterned distribution of dividing cells that is maintained during elongation. We classified this temporally stable spatial pattern as type 1, and it seems that it always correlates with the expression of segmentation genes in the SAZ, as in *Oncopeltus* and *Thamnocephalus*^19,20^. Something similar is found in the parasitoid wasp *Nasonia vitripennis*, but at later stages, within trunk segments that were already established^32^. Other insects, such as the beetles *Dermestes maculatus* and *Tribolium*, as well as *Drosophila*, display patterns of cell proliferation that change over time but are constant between individuals and are temporarily located in the posterior region of the extending germband. These predictable changing spatial and temporal patterns^7,33^ were classified as type 2 and showed no coincidence with segmentation genes expression patterns. Third, arthropods such as the centipede *Strigamia maritima* (Myriapoda) and the spider *Parasteatoda tepidariorum* (Chelicerata) show cell proliferation but without any recognizable spatial or temporal patterns (type 3; no pattern) during the formation of the thoracic and thoracic/opisthosomal segments, respectively^10,11^. The same is observed for the branchiopod crustacean *Artemia franciscana*^31,34^, the cockroach *Periplaneta americana*^35,36^, the cricket *Gryllus bimaculatus*^37^ and the polyembryonic wasp *Macrocentrus cingulum*^38^, where an unpatterned large number of dividing cells along the posterior trunk and growth zone were found at least during part of their axial elongation. If anything, given that most data concerning cell division patterns in these cases were obtained from indirect evidence, we cannot rule out the possibility that a more detailed analysis of the spatial and temporal distributions of cell divisions in these—and other—arthropods could reveal similarities with the type 1 or type 2 patterning mentioned before.

Considering these findings, it is easy to wonder if there is a relationship between the type of cell division patterning and its role during elongation. The constant low levels of cell proliferation found in *Oncopeltus* and *Thamnocephalus* appear as a continuous source of new cells that are refilling the SAZ as new segments form, with no apparent relation to any specific stage^19,20^. Compared to this, our results showed that cell proliferation patterns in *Tribolium* were temporally nonuniform and characterized by a peak of cell divisions taking place during thoracic segments formation. Given that this peak was also posteriorly localized, it is interesting that our analysis showed that nuclear compaction remained practically uniform and stable during elongation. The most likely explanation underlying this result is that cellular density did not increase in the SAZ—and to a lesser extent in the trunk—because the tissue was expanding.

We propose that the increase in the number of dividing cells drives the acceleration of elongation observed during the formation of thoracic segments. In turn, since the segmentation clock depends on the periodic transcription of cyclic genes^39–42^ and mitotic cells have low global transcriptional levels^43,44^, this would explain the slowing down of the segmentation rate found by Nakamoto et al*.*^23^ during the period where we found high proliferation. However, the reduction in the number of proliferating cells after the peak was not abrupt enough to account for the subsequent rise in the segmentation clock rate at the transition between thoracic and abdominal segments formation described by Nakamoto et al*.*^23^. What is triggering this shortening of the period of the segmentation clock—and the consequent acceleration in segment formation—remains unclear. Notably, convergent extension through mediolateral cell intercalation has been shown to control elongation, at least during the first half of abdominal segmentation^23,45^. At the same time, this could explain why abdominal segments tend to be much narrower than the thoracic segments.

The link that we found between the increase in cell proliferation during thoracic segments formation and thoracic segment size in turn seems to be connected to the increase in the rate of elongation during this period because the effect of inhibiting DNA synthesis by aphidicolin was larger on the length than on the width of thoracic segments. If we add the modest but significant contribution that anteriorly–posteriorly-oriented cell divisions must have on the elongation process, these results strongly support our proposal that elongation velocity depends, at least in part, on the number of cell divisions that take place during the corresponding period of germband extension.

Bearing in mind that *Tribolium* cell proliferation patterning is not uniform over time and space and is probably combined during elongation with coordinated cell movements such as convergent extension, it is interesting to note that in previous studies, the inhibition of both cellular processes results in disrupted posterior growth. When *Tribolium* embryos were incubated or injected with aphidicolin and hydroxyurea to block cell division, germbands exhibited impaired axial elongation or axial truncation, respectively, revealing that at least in the beetle *Tribolium*, cell proliferation is necessary for full elongation^21^. On the other hand, Benton et al*.*^46^ demonstrated that *even-skipped*-dependent cell rearrangements were indispensable for *Tribolium* axial elongation. This was confirmed by Nakamoto et al*.*^23^ who also showed that *even-skipped* RNAi injection did not affect cell proliferation, indicating that cell division and cell rearrangements are most likely differentially regulated in *Tribolium*.

Very recently, Benton et al*.*^25^ proposed that Toll genes play an ancient role in convergent extension/cell intercalation within Arthropoda and that they are probably regulated by *even-skipped* (*Tc-eve*) in *Tribolium* embryos. Given that *Tc-eve* is not involved in the regulation of cell division, as we commented above, it is striking that simultaneous knockdown of the two Toll genes expressed during the elongation of the spider *Parasteatoda* resulted in wider embryos without affecting their length and segment formation^25^. Without cell intercalations, this phenotype was only achievable by an important increase in cell divisions, suggesting that Toll genes in *Parasteatoda* are probably regulating both cellular processes, fostering convergent extension and restraining cell proliferation. By contrast, double knockdown of *Tribolium* Toll genes *Tc-Tl7* and *Tc-Tl10* caused shorter embryos without affecting segment specification^25^, revealing that the ancestral role of Toll genes in axial elongation was probably regulating the contribution of both cellular processes.

So what controls the variable proliferation levels over time seen in *Tribolium* and other arthropods with the type 2 cell division pattern? Contrary to that in *Tribolium*, *eve* RNAi injection in the beetle *Dermestes* resulted, in addition to shorter or truncated embryos, in almost completely elimination of the posteriorly localized high levels of mitotic cells that are normally found in the SAZ of control embryos^33^. This kind of evidence makes it unlikely that regulatory factors similar to those described above control cell proliferation during *Tribolium* elongation.

Recently, by RNA-seq after RNAi against Wnt/β-catenin pathway members, Oberhofer et al.^47^ identified several Wnt downstream genes that are required in the SAZ for patterning, mesoderm formation and cell division. Although there is no direct evidence for reduced cell proliferation after RNAi-mediated functional analysis of Wnt signaling in *Tribolium*^48–50^, our own preliminary data on blocking the Wnt pathway using pharmacological inhibitors (Mundaca-Escobar et al.,* unpublished observations*) during axial elongation showed fewer PH3^+^ cells than in the controls after a 2-h incubation. These findings are supported by previous results in cockroaches^36^, but not in spiders^51^.

Taken together, all this evidence reveals evolutionary plasticity at the level of the cellular processes involved in posterior growth and in their regulatory factors, showing great variability among arthropods. We propose that these highly variable patterns of cell division and cell movements may account for some of the substantial morphological diversity shown by arthropods.

## Methods

### Embryo collection

All embryos used in this work were offspring from the EFA-nGFP *Tribolium castaneum* transgenic line that ubiquitously expresses nuclear-localized GFP^40^. Stages were defined as minutes post horseshoe stage (mph)^46^. The horseshoe stage corresponds to the moment when the posterior amniotic fold extends anteriorly, covering part of the germband and forming a recognizable horseshoe-shaped amnion cover. All embryos were dissected at this stage (0 mph) and incubated as in Macaya et al*.*^52^ and posteriorly fixed in 4% formaldehyde at the desired stage, according to each experiment.

### Immunohistochemistry

Immunostaining was performed according to Shippy et al*.*^53^. Rabbit anti-phospho-Histone H3 pSer10 (PH3) (1:500, SIGMA H0412) was used as primary antibody and Donkey Anti-Rabbit IgG H&L coupled to Alexa Fluor 594 (1:500, ABCAM ab150076) as secondary antibody. Nuclei were visualized using ProLong Gold mounting medium with DAPI (MOLECULAR PROBES P36935). Embryos were flat mounted in a dorsal view and photographed (10×) using a Biotek Cytation 5 Cell Imaging Multi-Mode Reader.

### In situ hybridization

Embryos were washed three times in phosphate-buffered saline 1% (PBS), dehydrated with a methanol/PBT gradient (25, 50, 75, and 100%), and kept at 4 °C until used for in situ hybridization. Embryos were then rehydrated through a methanol/PBT gradient (75, 50 and 25%) and processed for hybridization and detection^53^ using *Tc-engrailed* digoxigenin-labeled RNA probe from Cepeda et al*.*^21^. Samples were photographed using a NIKON Eclipse Ci epifluorescence microscope (20×).

### Aphidicolin treatment

Aphidicolin (APH, SIGMA A0781) 10 mM stock in DMSO was diluted to 250 µM in M3 + medium. For time window Aphidicolin treatments, dissected embryos were incubated with APH for 30 min and washed/incubated with M3 + medium according to the experiment design (Fig. 2a). Once incubation was completed, embryos were fixed and mounted for posterior immunostaining protocol. For the thoracic segments formation experiments, dissected embryos were incubated with APH for 1 h (between o and 60 mph) and then washed and incubated with M3 + medium for another 4 h. Once M3 + incubation was completed, embryos were fixed for posterior in situ hybridization protocol.

### Image and data analysis

All image analysis was performed using ImageJ (FIJI) software. For the proliferation pattern analysis, each PH3 immuno-stained embryo was photographed in different focal planes (9.9 µm each *z*-stack) with a maximum projection. Deconvolution was performed using the GEN5 IMAGE PRIME 3.08 software with standard deviation: 0.959; kernel Radius: 2px (3 × 3 matrix); Iterations: 5. PH3^+^ cells were manually counted using *Cell Counter* tool. The obtained values of PH3^+^ cells were normalized by embryo area and volume. Germband area was measured in binarized DAPI images without considering head lobes, and length was measured as in Macaya et al*.*^52^. Germband volume was calculated using the *Measure Stack* plugin for ImageJ, based on the individual focal planes area and the distance between *z*-stacks. Given that germband thickness appeared to remain relatively constant during elongation and that cell division patterning looks very similar after both area normalization and volume normalization (Supplementary Fig. S16), we decided to continue normalizing by germband area. Germband regions R1, R2 and R3 were established as a length of 140.2 µm each, starting from the posterior end but excluding the mesoderm tip. This size was used for all analyzed embryos and was determined according to *Tc-caudal* expression zone length which was previously measured in 0 mph embryos by in situ hybridization^21^. Segment size analysis was performed based on in situ hybridization images. Length and width were measured at the center of each analyzed segment (T1, T2, T3 and A1) using the *Straight Line* tool. For area measurement the perimeter of each segment, delimited by *engrailed* stripes, was selected and the cropped zone was binarized. For APH embryo images, *Retinex filter* was previously used to distinguish the limit of segments (Supplementary Fig. S12). Angles of cell division were manually measured in immune-stained embryo images positioned vertically using *Angle tool* option in mitotic cells under metaphase or anaphase stage. Angles were classified in three groups with respect to the antero-posterior axis: (i) 0°–30° and 150°–180°, (ii) 30°–60° and 120°–150°, and (iii) 60°–120° (see Fig. 5b). For cell density analysis, embryos of each stage were photographed (20×) with a NIKON C1Plus confocal microscope, and nuclei were counted in an arbitrary area of 67.5 µm × 67.5 µm of dorsal tissue using the *Cell Counter* tool. Germband elongation rate was calculated as the slope from the linear regression of each stage length data. Elongation percentage and change rate were calculated considering the average length of 0 mph embryos (272.0 μm) as 0%, and the average length of 300 mph embryos (622.0 μm) as 100%.

### Statistical analysis

Data were analyzed using GRAPHPAD PRISM 8.0.2 version software. Data distributions and homoscedasticity were checked by D’Agostino-Pearson, Shapiro–Wilk and Brown–Forsythe tests. For data with Gaussian distribution and homoscedasticity, one or two-way ANOVA were performed for one or two categorical variables, respectively, followed by a Tukey multiple comparison post hoc test. Groups without homoscedasticity were compared with Tamhane’s T2 test. Groups without Gaussian distribution were compared with nonparametric Kruskal–Wallis test followed by a Dunn multiple comparison post hoc test. Regressions analyses were compared using F-test. Chi-square tests were made by R Studio software. Different letters represent groups with statistically significant differences (*p* < *0.05*). Asterisks indicate statistical significance of: *p < 0.05, **p < 0.01, ***p < 0.001 and ****p < 0.0001.

## Supplementary Information

Supplementary File.
