# Supplementary material for: Spatiotemporal variation in cell proliferation patterns during arthropod axial elongation
Source: Sci Rep. 2021 Jan 11;11:327. doi: 10.1038/s41598-020-79373-0 (PMC7801698; doi:10.1038/s41598-020-79373-0)
Supplement: Supplementary file 1 — Supplementary File. [file 41598_2020_79373_MOESM1_ESM.pdf]

Supplementary Information

Spatiotemporal variation in cell proliferation patterns during arthropod axial elongation

Rodrigo E. Cepeda[#], John B. Terraza[#], Renato V. Pardo, Valentina Núñez-Pascual, Marco Mundaca-Escobar, Andres F. Sarrazin

Instituto de Química, Pontificia Universidad Católica de Valparaíso
Valparaíso, Chile

Supplementary Figures

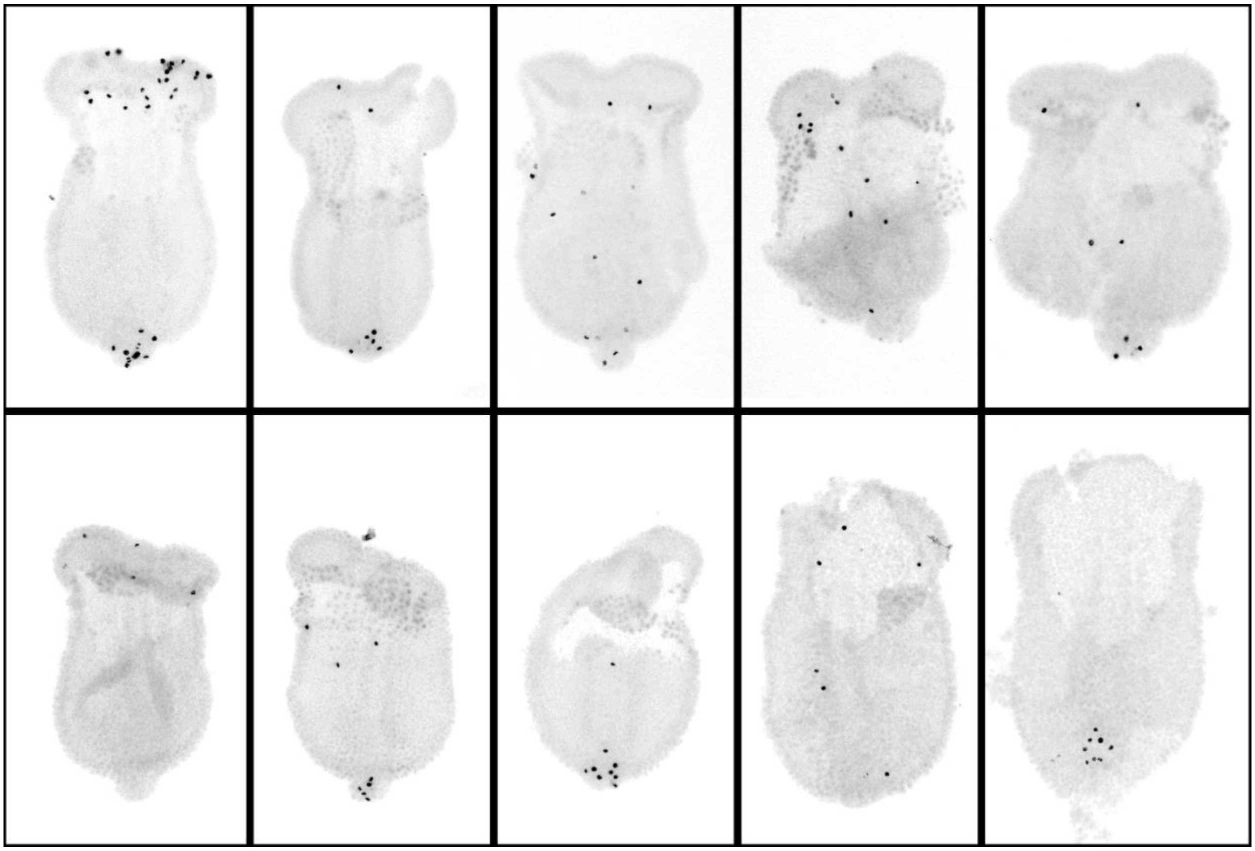

Supplemental Figure S1. PH3 immunostaining (mitotic marker) of all germbands analyzed at 0 mph. Red fluorescent images were converted to black and white and color-inverted to improve visualization. All embryos showed are dorsally oriented. Anterior is to the top.

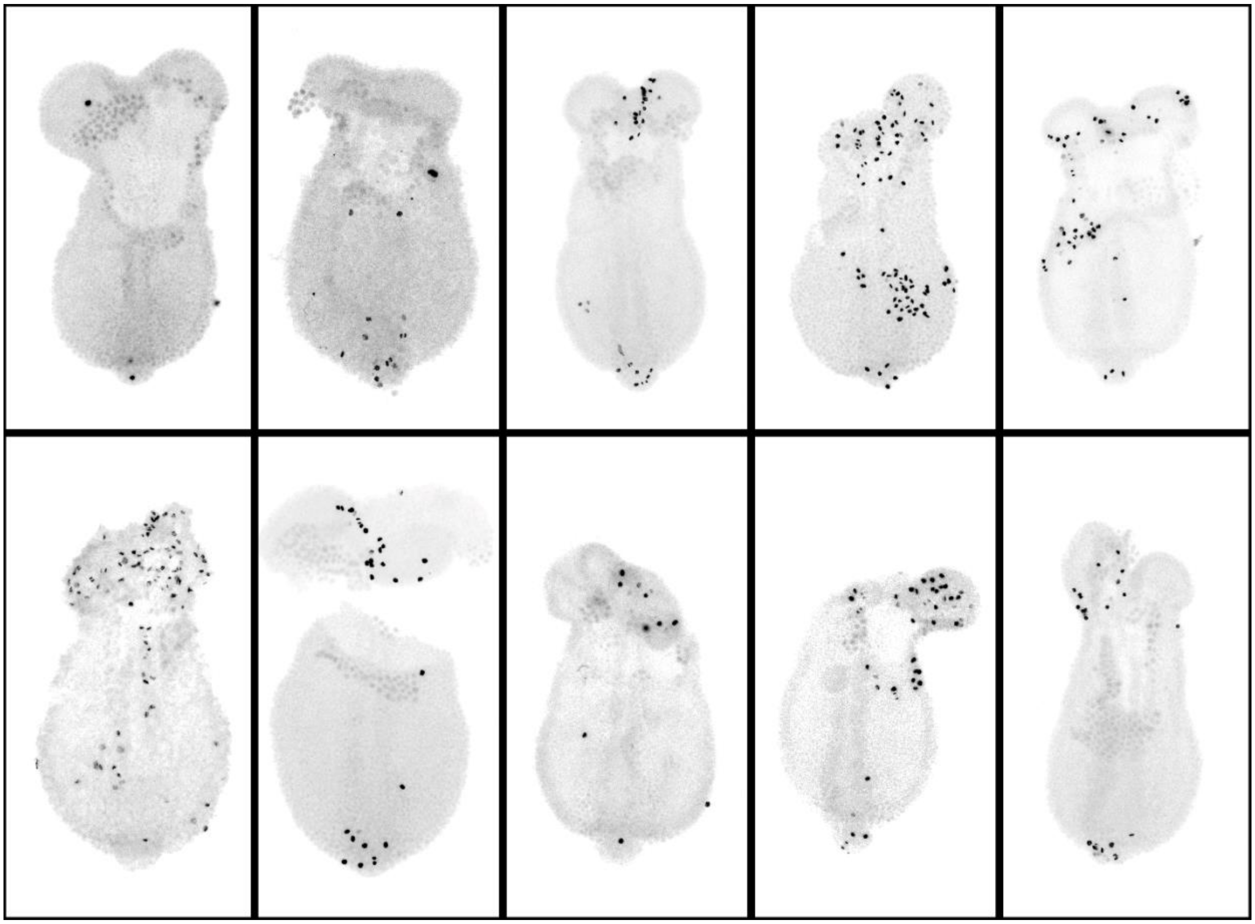

Supplemental Figure S2. PH3 immunostaining (mitotic marker) of all germbands analyzed at 30 mph. Red fluorescent images were converted to black and white and color-inverted to improve visualization. All embryos showed are dorsally oriented. Anterior is to the top.

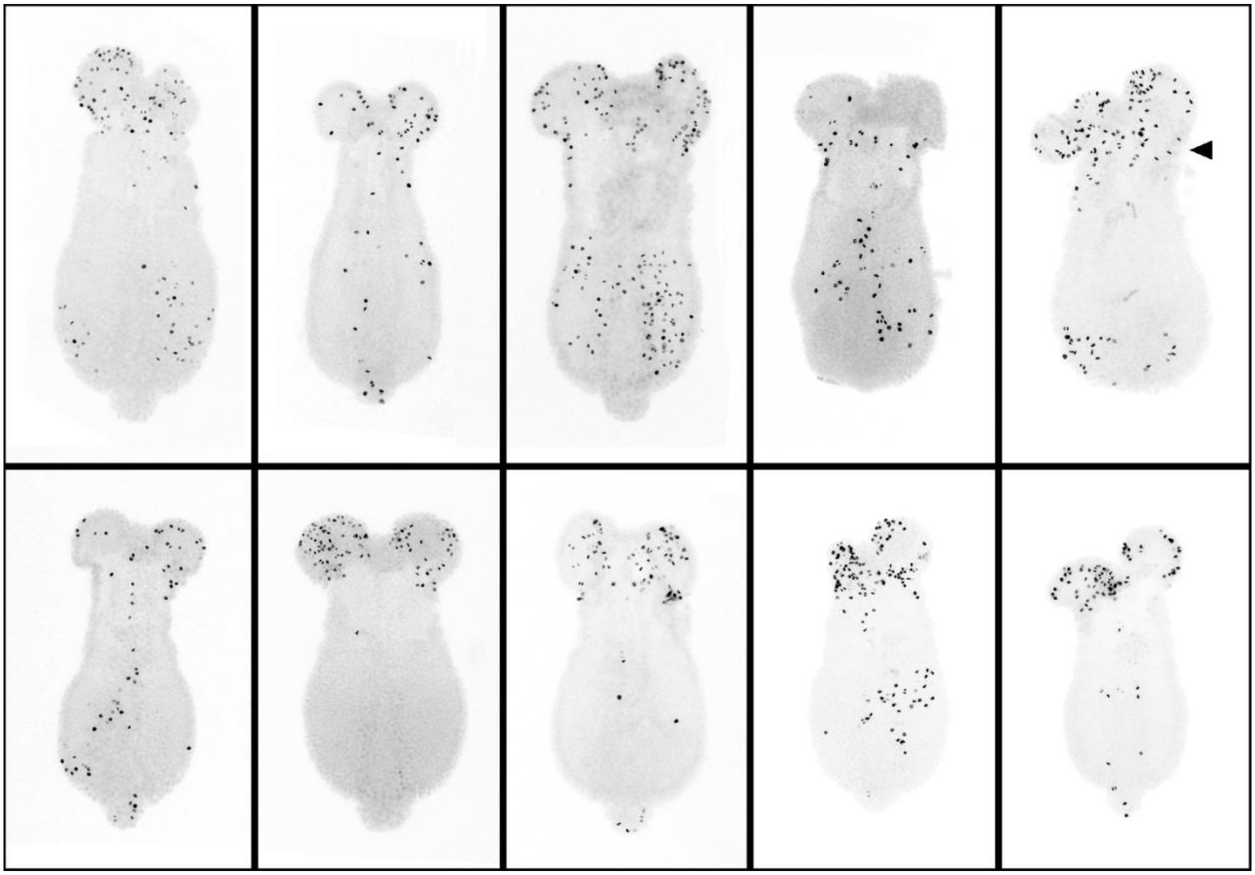

Supplemental Figure S3. PH3 immunostaining (mitotic marker) of all germbands analyzed at 60 mph. Red fluorescent images were converted to black and white and color-inverted to improve visualization. Arrowhead indicates the base of the head lobes when it is not evident. All embryos showed are dorsally oriented. Anterior is to the top.

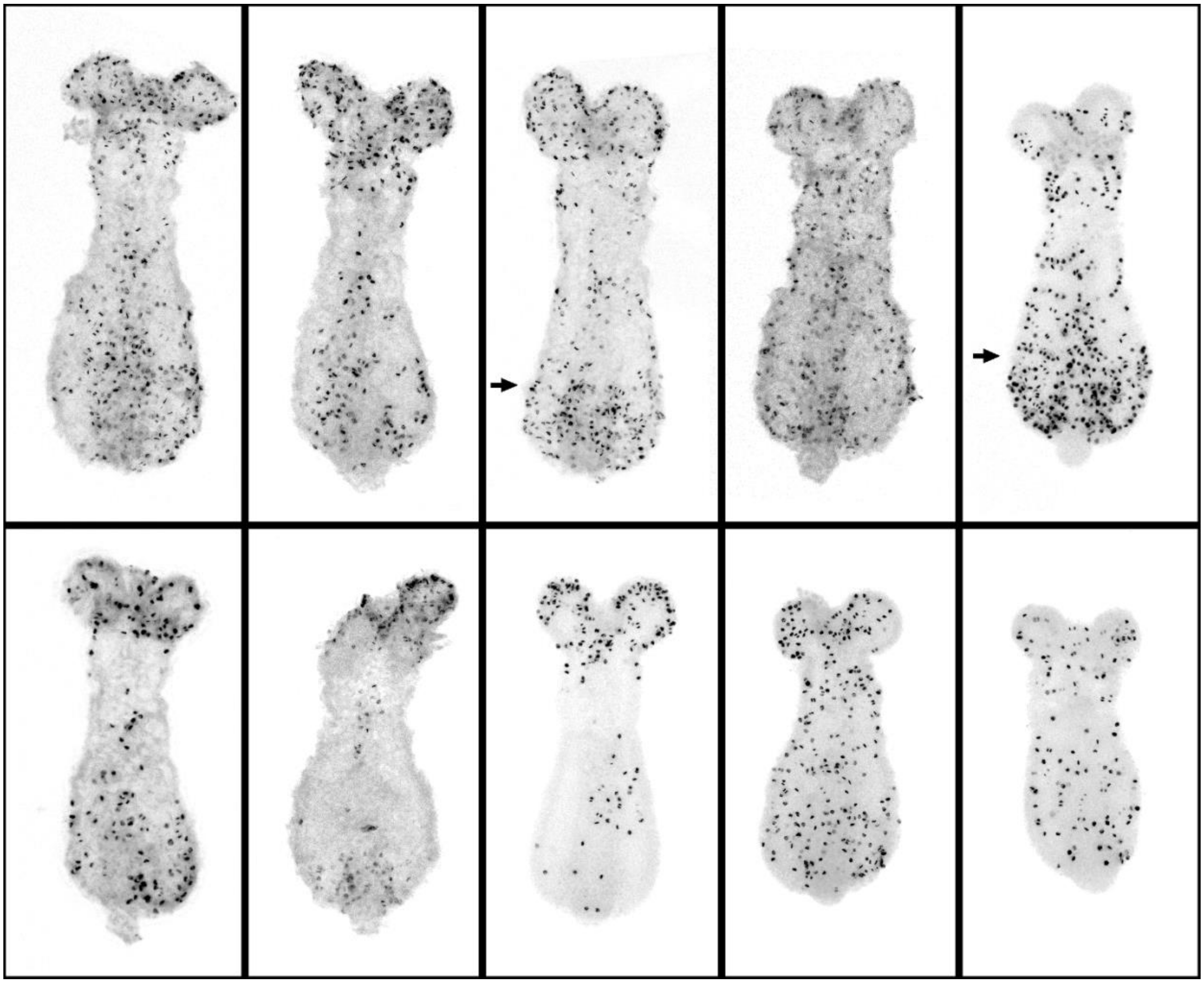

Supplemental Figure S4. PH3 immunostaining (mitotic marker) of all germbands analyzed at 90 mph. Red fluorescent images were converted to black and white and color-inverted to improve visualization. Arrows point to the high degree of $PH3^{+}$ cells at the SAZ. All embryos showed are dorsally oriented. Anterior is to the top.

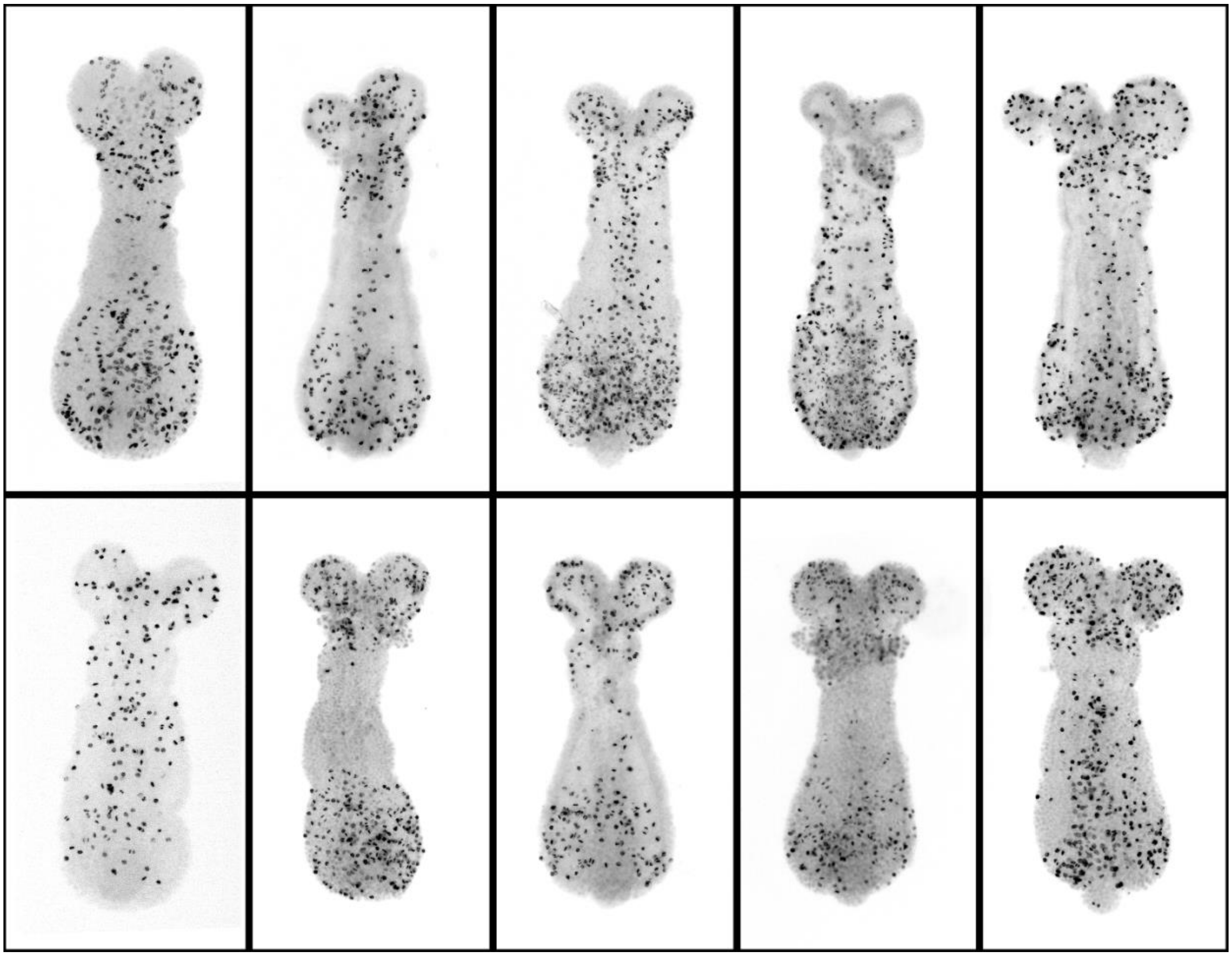

Supplemental Figure S5. PH3 immunostaining (mitotic marker) of all germbands analyzed at 120 mph. Red fluorescent images were converted to black and white and color-inverted to improve visualization. All embryos showed are dorsally oriented. Anterior is to the top.

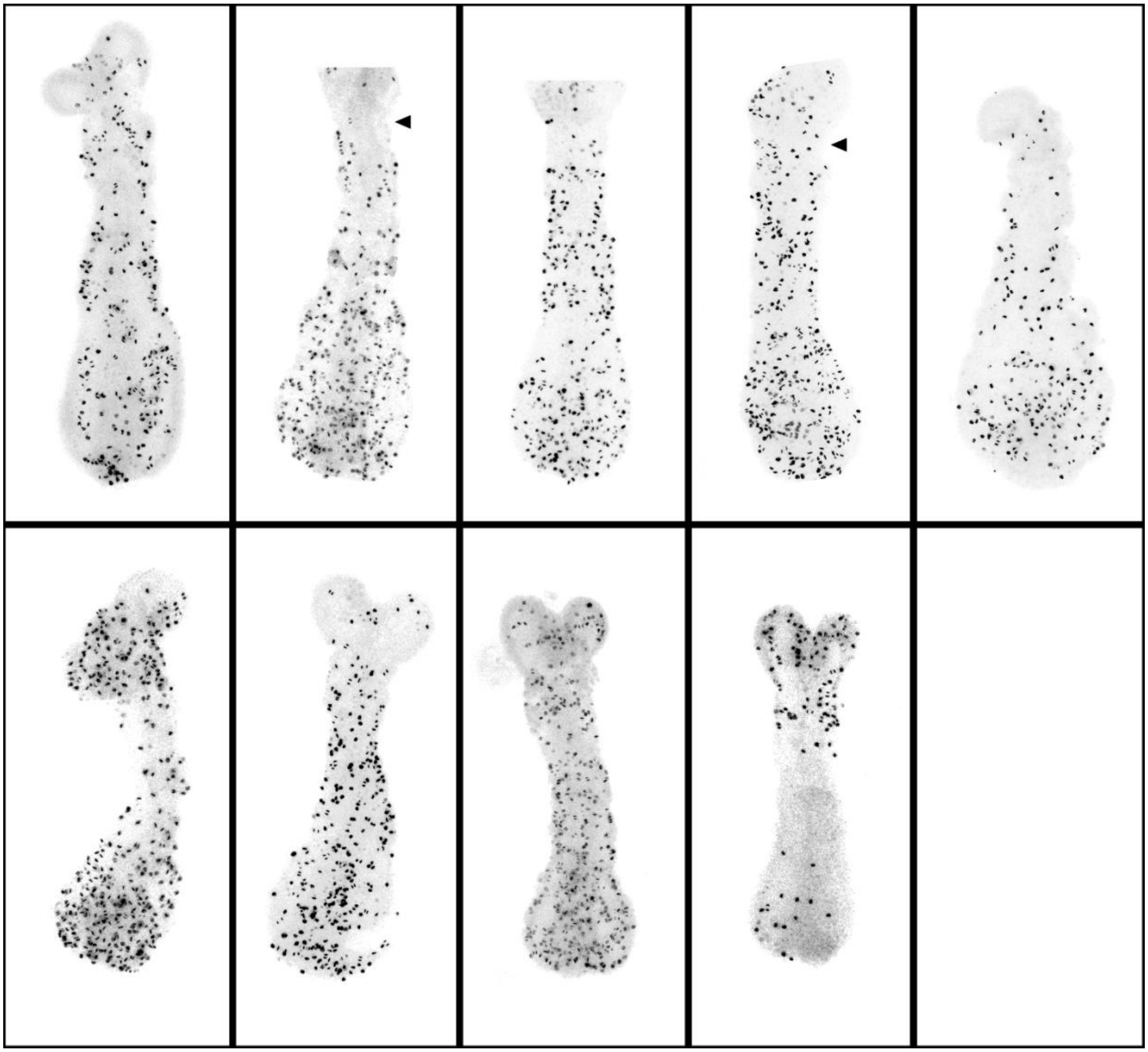

Supplemental Figure S6. PH3 immunostaining (mitotic marker) of all germbands analyzed at 150 mph. Red fluorescent images were converted to black and white and color-inverted to improve visualization. Arrowhead indicates the base of the head lobes when it is not evident. All embryos showed are dorsally oriented. Anterior is to the top.

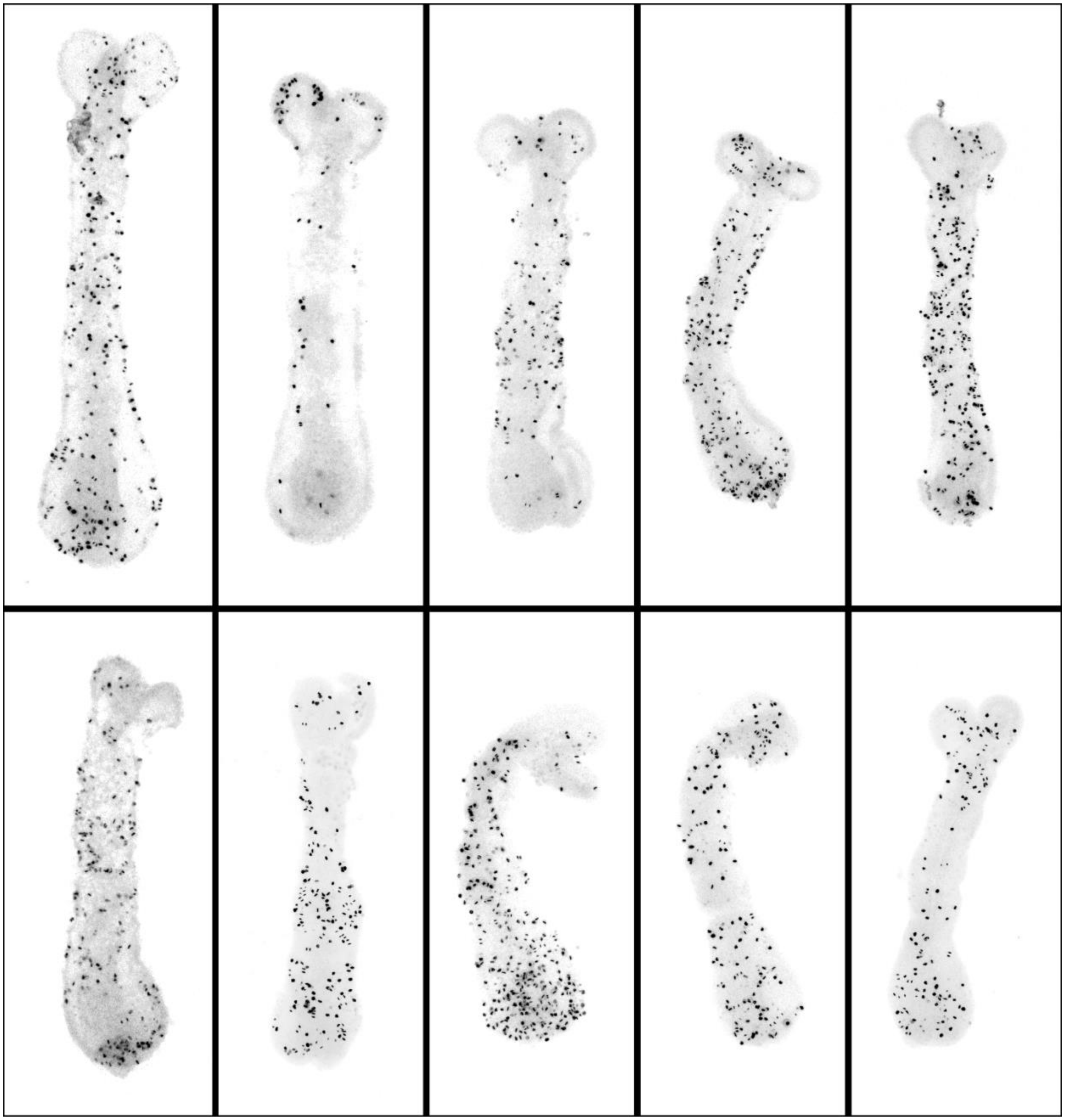

Supplemental Figure S7. PH3 immunostaining (mitotic marker) of all germbands analyzed at 180 mph. Red fluorescent images were converted to black and white and color-inverted to improve visualization. All embryos showed are dorsally oriented. Anterior is to the top.

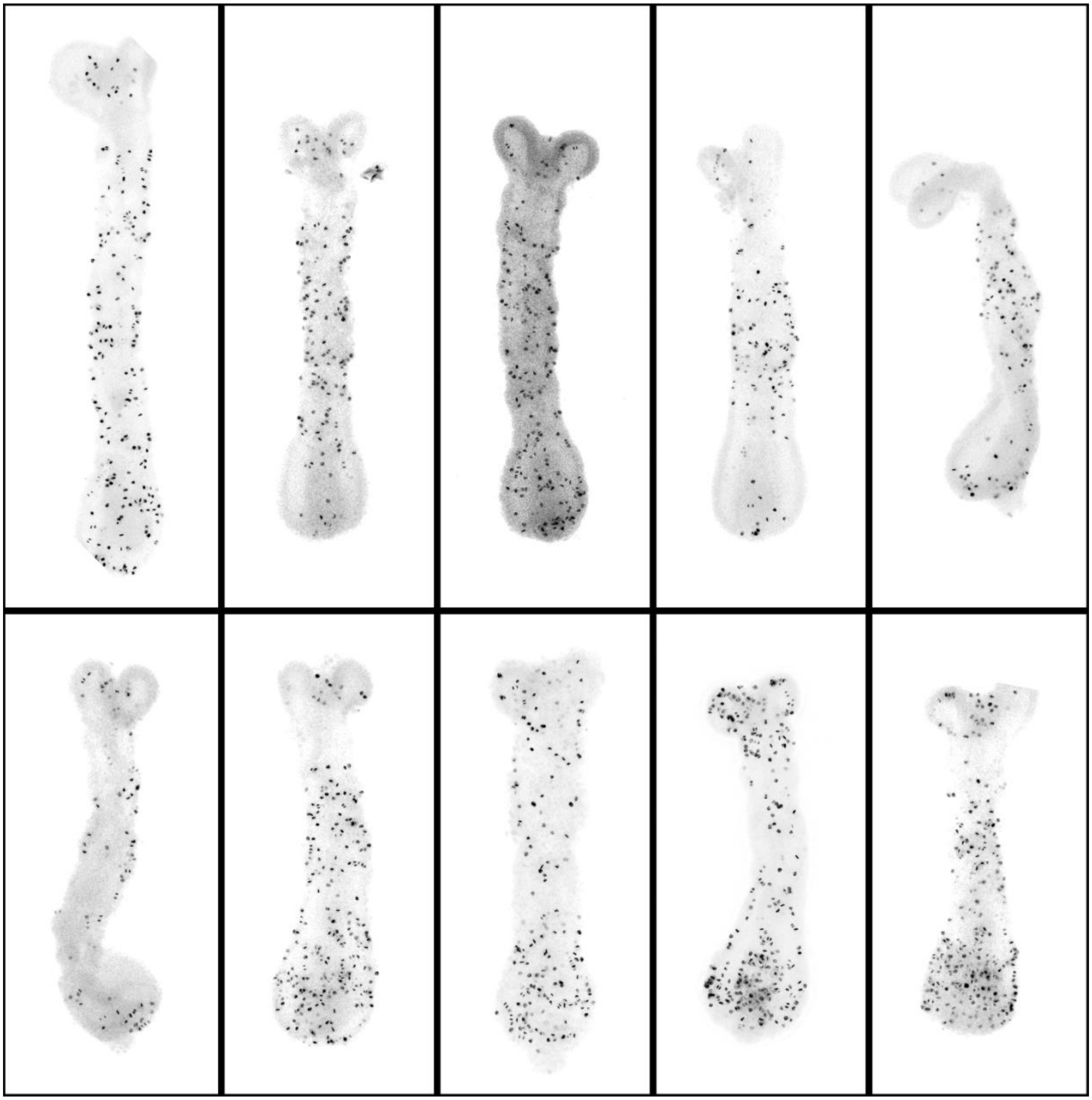

Supplemental Figure S8. PH3 immunostaining (mitotic marker) of all germbands analyzed at 210 mph. Red fluorescent images were converted to black and white and color-inverted to improve visualization. All embryos showed are dorsally oriented. Anterior is to the top.

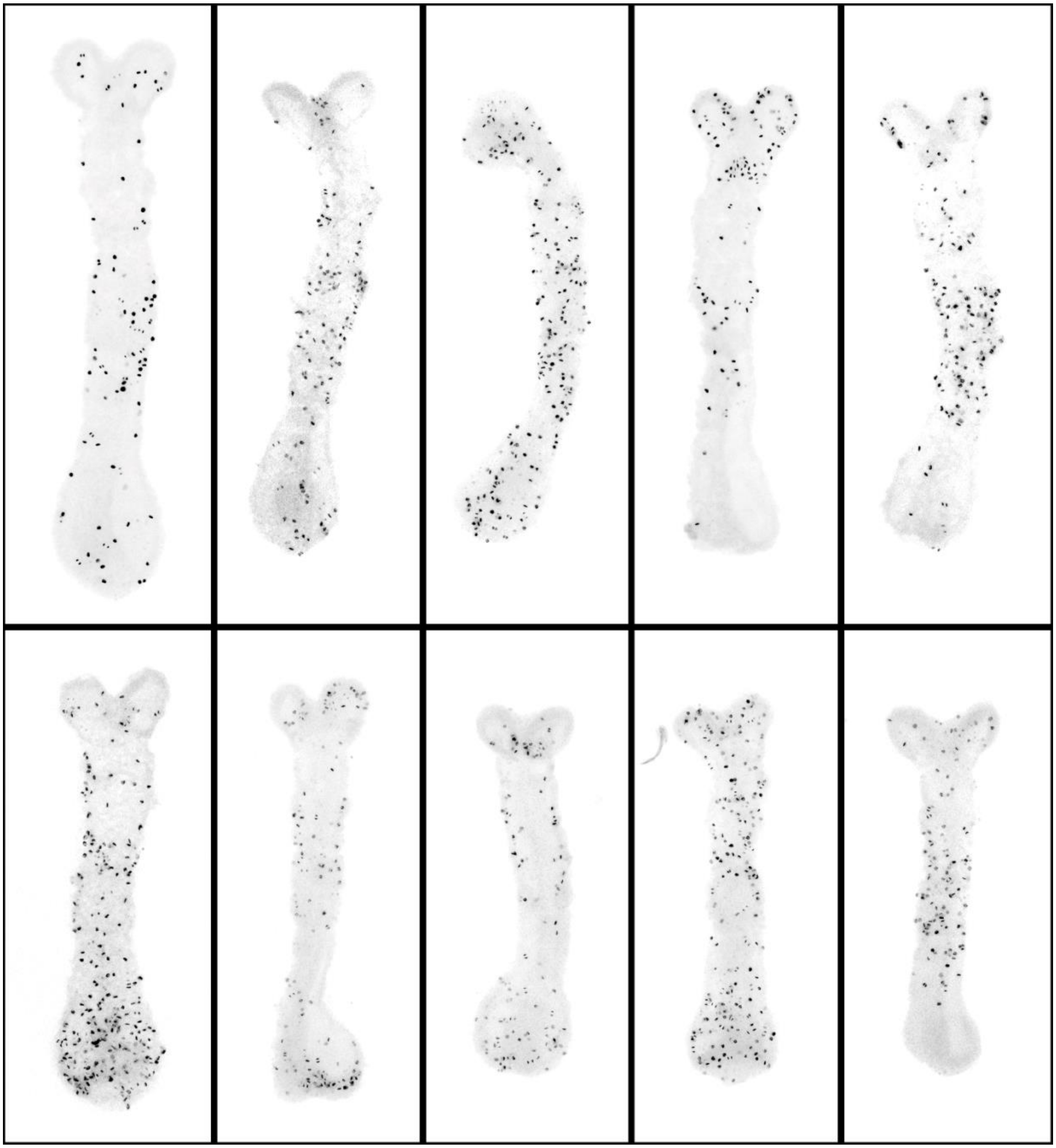

Supplemental Figure S9. PH3 immunostaining (mitotic marker) of all germbands analyzed at 240 mph. Red fluorescent images were converted to black and white and color-inverted to improve visualization. All embryos showed are dorsally oriented. Anterior is to the top.

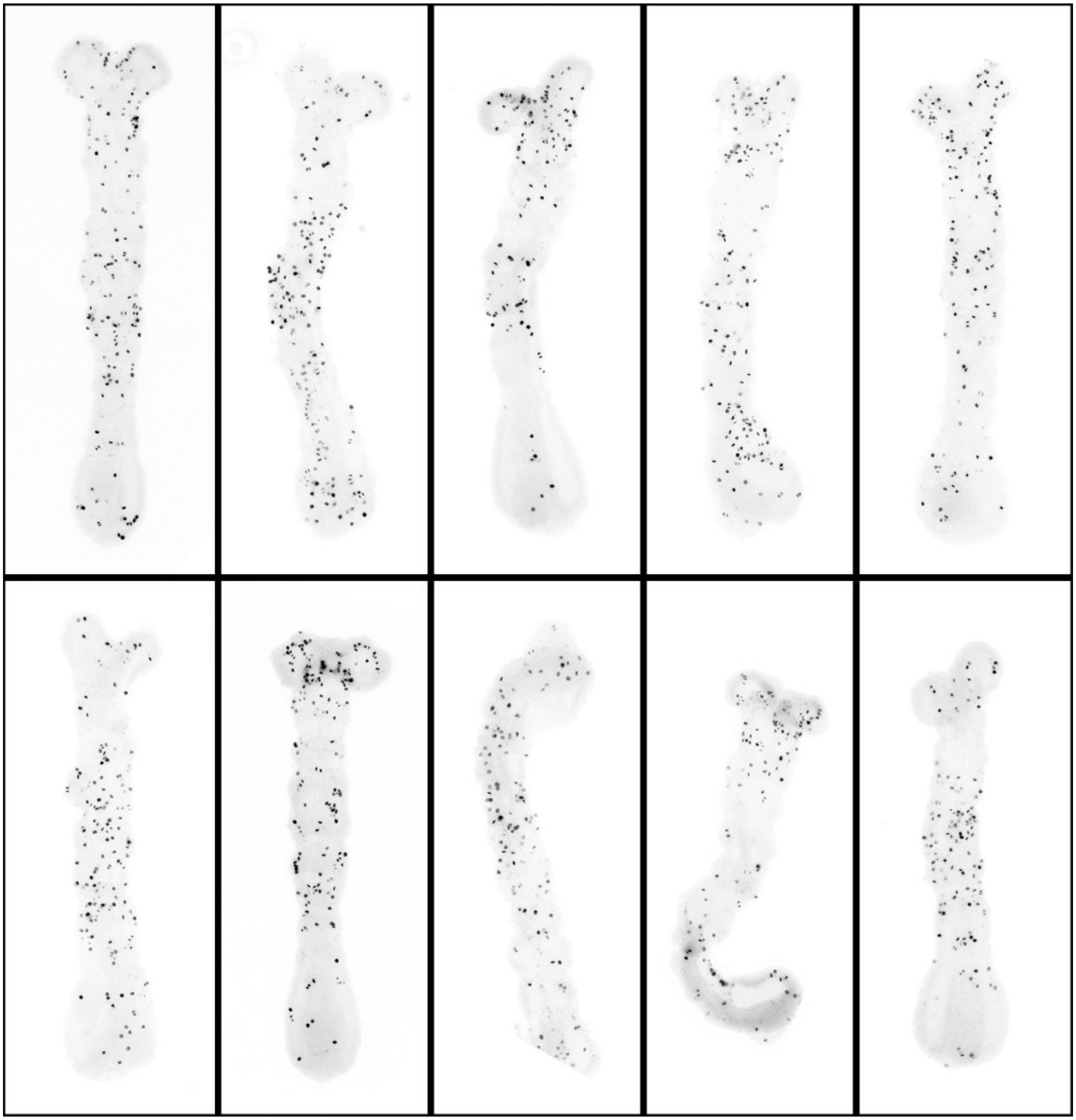

Supplemental Figure S10. PH3 immunostaining (mitotic marker) of all germbands analyzed at 300 mph. Red fluorescent images were converted to black and white and color-inverted to improve visualization. All embryos showed are dorsally oriented. Anterior is to the top.

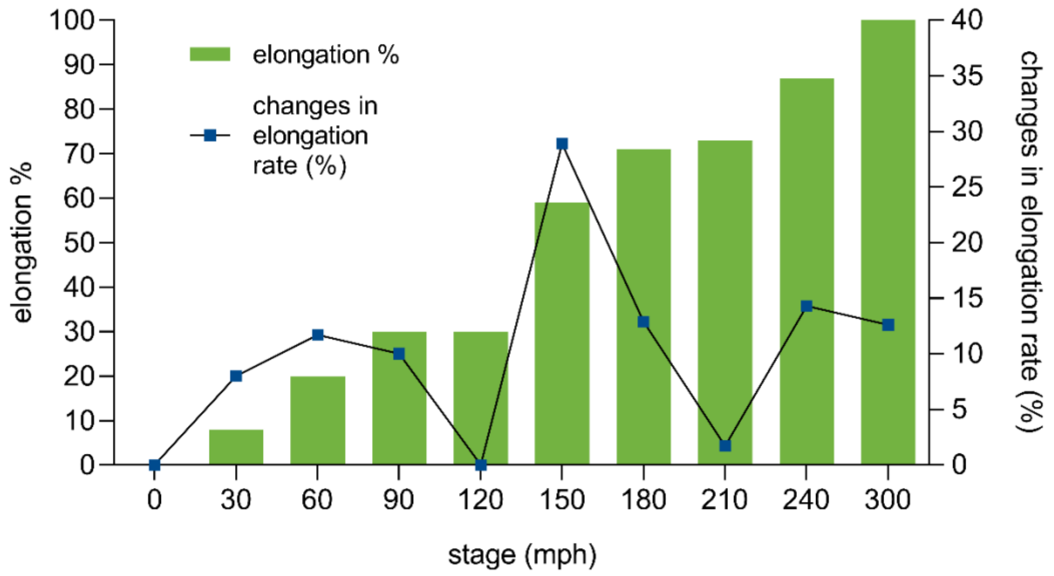

Supplementary Figure S11. Changes in the elongation rate and percentages of elongation by stage. Elongation percentage (bars) and percentage of change in the elongation rate (blue squares within the curve) analysis during the whole period quantified showed a sudden leap in the elongation rate from 120 to 150 mph that allowed us to identify an increase in the elongation rate at the 120-180 mph period compared with the periods just before and after (Fig. 3b).

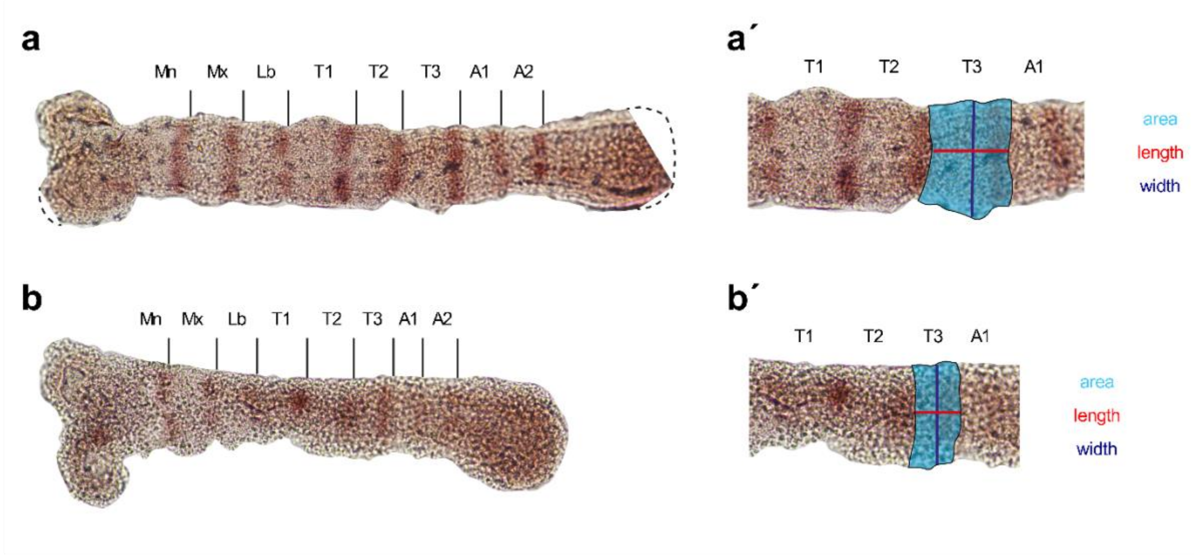

Supplementary Figure 12. Segment size measurement. (**a,b**) Representative pictures (n = 5) of control (**a,a'**) and aphidicolin treated (**b,b'**) germbands stained with a *Tc-engrailed* riboprobe in order to evaluate the area (light blue), length (red line) and width (blue line) of the three thoracic (T1, T2 and T3) and first abdominal (A1) segments. (**a',b'**) Higher magnification showing an example in one segment (T3) of the three different measurements performed. Segments from anterior to posterior: Mn: Mandibular; Mx: Maxillary; Lb: Labial; T1-T2-T3: Thoracic 1, 2 and 3; A1-A2: Abdominal 1 and 2. Dorsal views. Anterior is to the left.

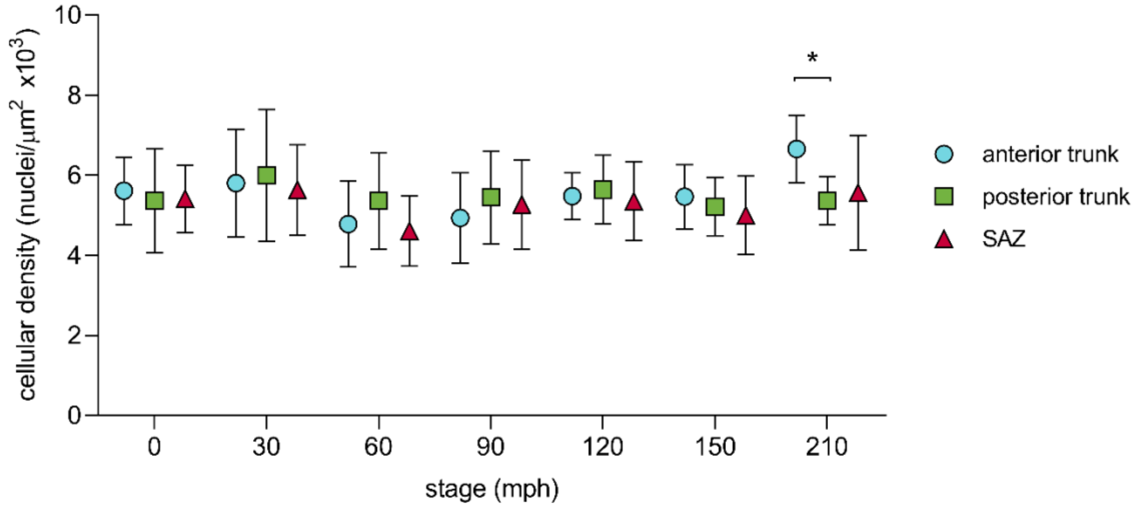

Supplementary Figure S13. Spatial and temporal measurements of cellular density. Statistical analysis of the quantity of cellular nuclei by area (as a meaning of cellular compaction) along the extending germband at different stages (0 – 210 mph) and positions (anterior trunk, posterior trunk and the segment addition zone). Error bars represent SD (n = 3 - 4). Asterisk indicate statistically significant differences according to ANOVA test of time-averaged data ($p = 0.0332$).

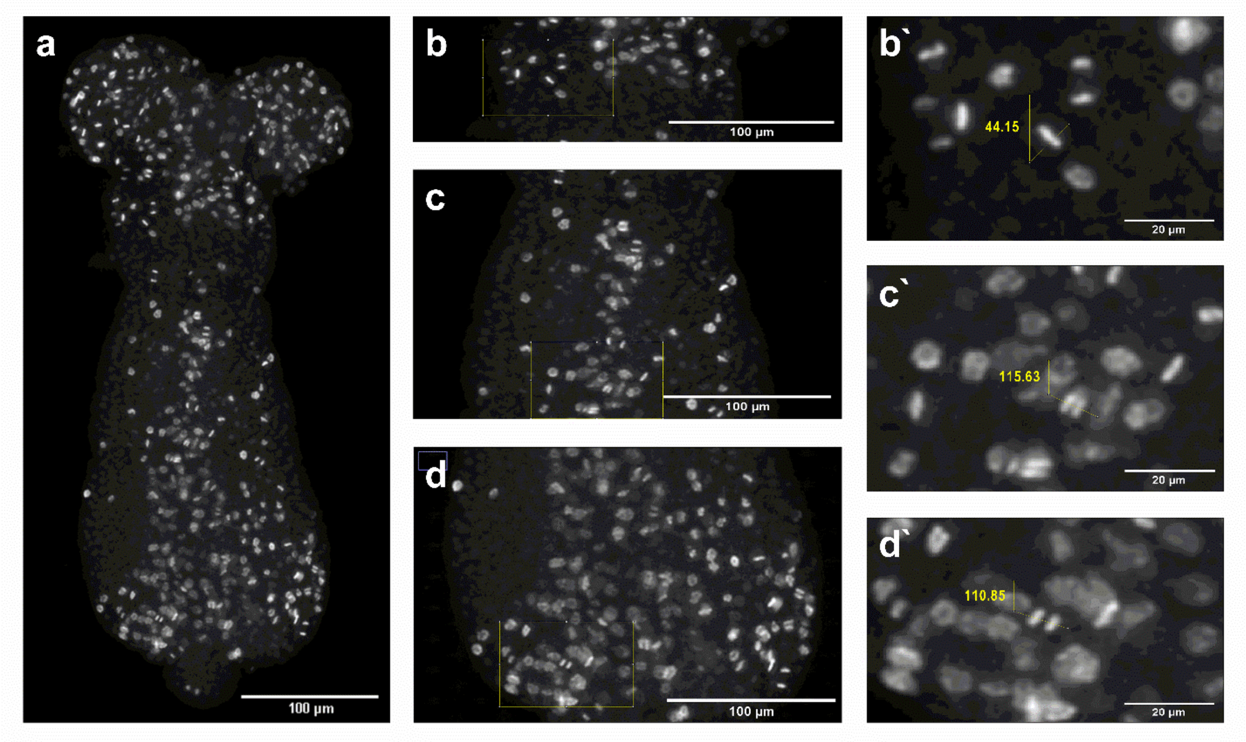

Supplementary Figure S14. Mitotic angles measurement. (**a**) Representative picture of an embryo stained with the fluorescent antibody anti-PH3 in order to show dividing cells in the germband during elongation (red fluorescent images were converted to black and white to improve visualization). (**b,c,d**) In each embryo, all PH3-labelled cells in metaphase and anaphase (~ 20 % of the total $PH3^+$ cells) along the germband were selected and their angles measured. Here we show three selected regions at different positions along the germband. (**a',b',c'**) Examples of one cell from each selected region (yellow square) for the analysis of the mitotic angle relative to the elongation axis (vertical line; 0°).

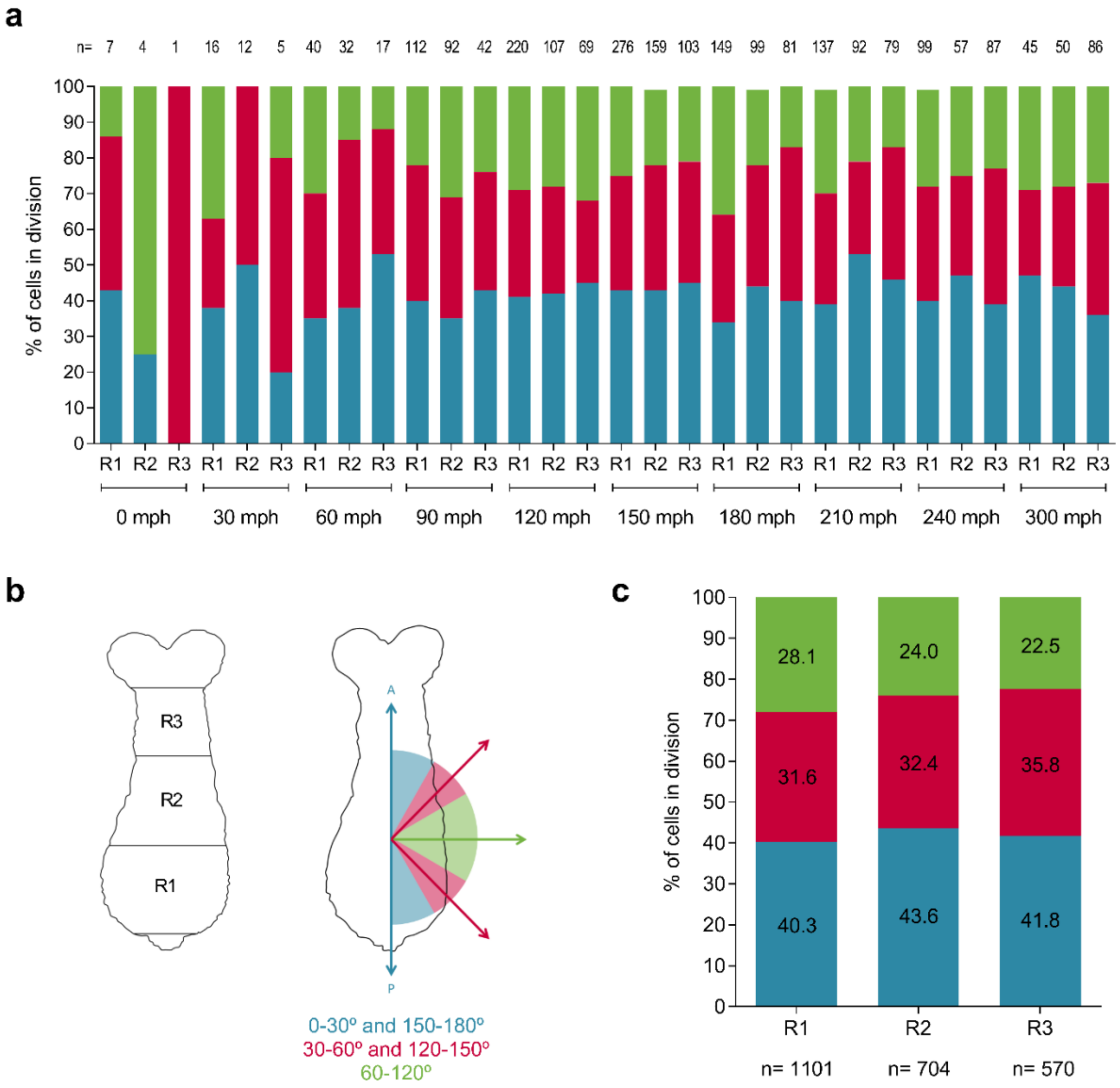

Supplementary Figure S15. Cell division orientation analyzed by region. (**a**) Percentage distribution of mitotic angles relative to the anterior-posterior axis (elongation axis) by stage and region (R1, R2, R3). (**b**) Schematic diagrams of a *Tribolium* germband subdivided in the 3 consecutive regions analyzed (left) and showing the angle selection criteria and color code (right). Blue: mitotic angles between 0° – 30° and 150° – 180°; Red: 30° – 60° and 120° – 150°; Green: 60° – 120°. (**c**) Total percentage distribution of mitotic angles relative to the anterior-posterior axis, grouped by region (R1, R2, R3). The bars represent the percentage of dividing cells that showed the corresponding angle range at each region. Number of cells analyzed (n) is also shown in (**a**) and (**c**).

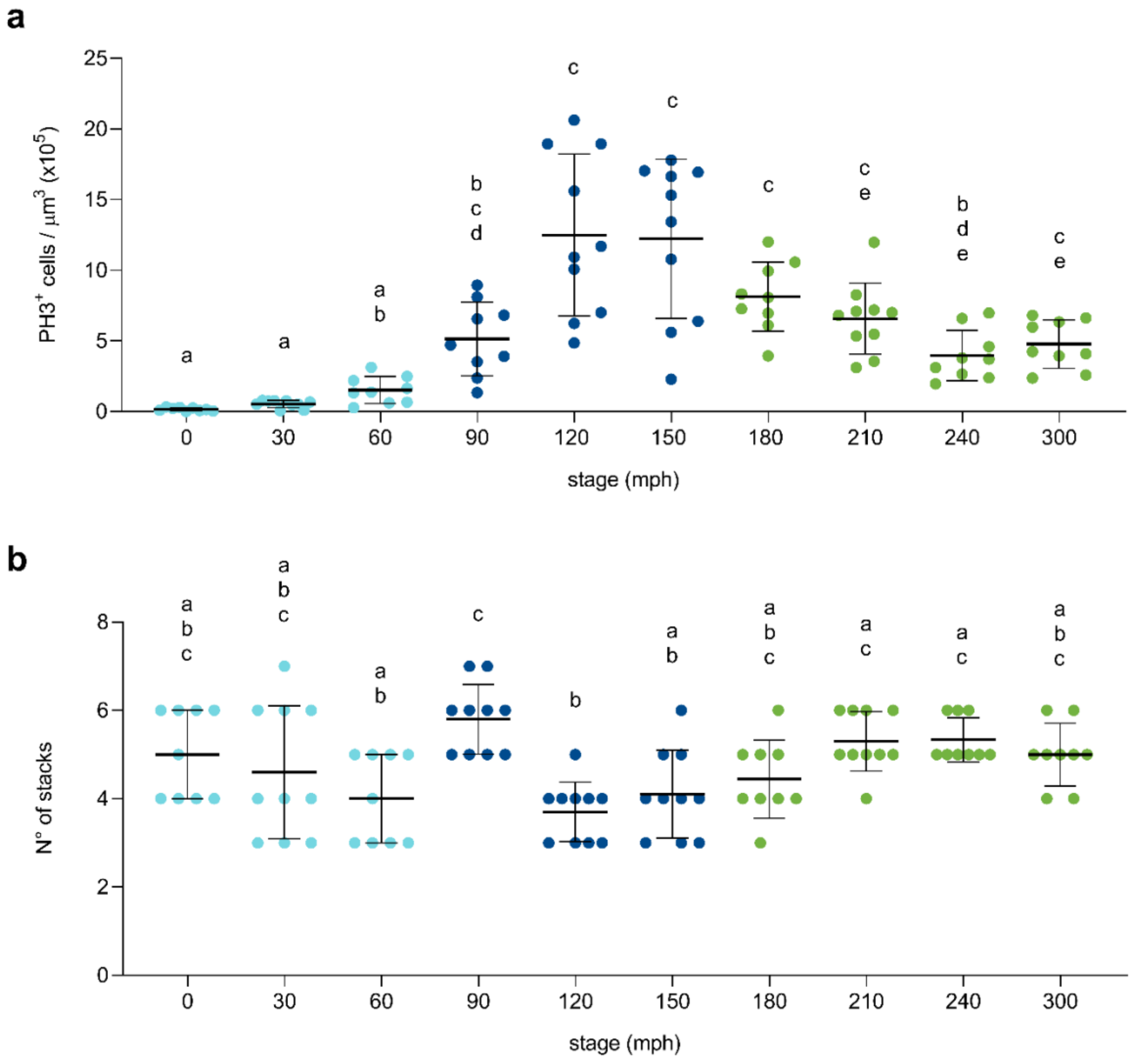

Supplementary Figure S16. PH3 data normalized by germband volume. (**a**) PH3$^+$ cells quantification (normalized by volume) every 30 minutes from 0 mph to 300 mph (n = 9-10; 270 mph was not counted). Temporal patterning of cell division during elongation was practically identical after both area normalization (**Fig. 1a**) and volume normalization. (**b**) Number of *z*-stacks photographed in each embryo, used as a parameter to calculate germband volume. Note that embryo thickness remains relatively constant during elongation. Different letters represent groups with statistically significant differences according to (**a**) a Brown-Forsythe and Welch ANOVA test ($p < 0.05$) and (**b**) a Kruskal-Wallis test and Dunn's multiple comparisons test ($p < 0.05$).

Supplementary Tables

Stage (mph)	0	30	60	90	120	150	180	210	240	300
Germband (total amount)	70	185	422	1437	2539	2710	1590	1482	1212	1140
Germband (mean ± SD)	7.0 ± 3.5	18.5 ± 8.8	42.2 ± 28.2	143.7 ± 77.8	253.9 ± 101.4	271.0 ± 91.4	159.0 ± 71.7	148.2 ± 49.4	121.2 ± 51.6	114.0 ± 24.2
Normalized by area	0.13 ± 0.07	0.26 ± 0.16	0.73 ± 0.48	2.82 ± 1.48	5.12 ± 1.96	4.90 ± 1.81	3.29 ± 1.59	2.85 ± 0.85	2.39 ± 11.14	2.11 ± 0.42
Normalized by volume	0.16 ± 0.12	0.54 ± 0.28	1.52 ± 0.95	4.94 ± 2.54	12.49 ± 5.73	12.22 ± 5.63	8.14 ± 2.45	6.58 ± 2.51	3.98 ± 1.78	4.78 ± 1.71
R1 (mean ± SD)	3.6 ± 2.8	5.9 ± 4.3	14.0 ± 12.1	80.7 ± 43.3	146.5 ± 82.0	127.7 ± 51.2	65.4 ± 47.2	62.5 ± 38.3	35.4 ± 35.2	21 ± 12
Normalized by area	0.13 ± 0.11	0.21 ± 0.15	0.50 ± 0.42	3.34 ± 1.75	6.50 ± 3.72	5.77 ± 2.45	3.52 ± 2.35	3.44 ± 1.94	1.87 ± 1.76	1.35 ± 0.80
R2 (mean ± SD)	1.30 ± 1.57	4.7 ± 5.7	11.2 ± 11.1	38.4 ± 22.1	46.0 ± 29.9	72.3 ± 31.9	40.8 ± 22.0	38.5 ± 11.6	22.5 ± 14.1	24.2 ± 13.5
Normalized by area	0.05 ± 0.06	0.18 ± 0.24	0.44 ± 0.40	1.93 ± 1.07	2.47 ± 1.57	4.23 ± 1.98	2.93 ± 1.80	3.00 ± 0.87	1.79 ± 1.06	2.16 ± 1.11
R3 (mean ± SD)	0.5 ± 1.0	2.7 ± 3.1	8.6 ± 5.0	24.0 ± 15.9	34.2 ± 14.4	48.0 ± 18.5	36.5 ± 17.6	36.6 ± 12.1	34.3 ± 11.9	37.7 ± 13.4
Normalized by area	0.07 ± 0.13	0.51 ± 0.54	0.96 ± 1.06	2.32 ± 0.88	3.27 ± 1.09	3.76 ± 1.43	3.07 ± 1.67	3.07 ± 1.05	2.68 ± 0.94	3.29 ± 1.14

Supplementary Table S1. Proliferating cells (PH3^{+}) quantified by stage (temporal pattern) and region (spatial pattern). Proliferating cells labelled with anti-PH3 antibody at each stage analyzed (orange first row). Quantification (showed as the average ± standard deviation in white and normalized by the area or volume in grey) was performed in the whole germband (without considering head lobes, second row) and at three different positions along the main axis of the embryo (R1, R2 and R3 regions from posterior to anterior; third, fourth and fifth rows).

Figure	Compared groups	p-value	Figure	Compared groups	p-value
1a	0 mph vs. 90 mph	0,0122	4a	T1 APH vs. T1 Control	<0,0001
	0 mph vs. 120 mph	0,0009		T2 APH vs. T2 Control	0,0001
	0 mph vs. 150 mph	0,0007		T3 APH vs. T3 Control	0,0079
	0 mph vs. 180 mph	0,0063	4b	T1 APH vs. T1 Control	0,0079
	0 mph vs. 210 mph	0,0001		T2 APH vs. T2 Control	0,0054
	0 mph vs. 240 mph	0,0062		T3 APH vs. T3 Control	0,0009
	0 mph vs. 300 mph	<0,0001	4c	T1 APH vs. T1 Control	0,0039
	30 mph vs. 90 mph	0,0171	S16a	0 vs. 90	0,0195
	30 mph vs. 120 mph	0,0011		0 vs. 120	0,0035
	30 mph vs. 150 mph	0,0009		0 vs. 150	0,0037
	30 mph vs. 180 mph	0,0084		0 vs. 180	0,0004
	30 mph vs. 210 mph	0,0002		0 vs. 210	0,0009
	30 mph vs. 240 mph	0,0091		0 vs. 240	0,009
	30 mph vs. 300 mph	<0,0001		0 vs. 300	0,0017
	60 mph vs. 120 mph	0,0019		30 vs. 90	0,0316
	60 mph vs. 150 mph	0,0014		30 vs. 120	0,0044
	60 mph vs. 180 mph	0,0239		30 vs. 150	0,0046
	60 mph vs. 210 mph	0,0003		30 vs. 180	0,0006
	60 mph vs. 240 mph	0,0467		30 vs. 210	0,0014
	60 mph vs. 300 mph	<0,0001		30 vs. 240	0,0171
	120 mph vs. 300 mph	0,0369		30 vs. 300	0,0029
	150 mph vs. 300 mph	0,0352		60 vs. 120	0,0075
1c	R2 vs. R1 90 mph	0,0204		60 vs. 150	0,0079
	R3 vs. R1 120 mph	0,0252		60 vs. 180	0,0007
	R2 vs. R1 120 mph	0,0038		60 vs. 210	0,0035
	R2 vs. R1 150 mph	0,0176		60 vs. 300	0,0125
	R3 vs. R2 300 mph	0,0013		120 vs. 240	0,0429
	R3 vs. R1 300 mph	0,0007		150 vs. 240	0,0477
2b	Control 2h vs. APH 2h	<0,0001		180 vs. 240	0,0419
	Control 2h vs. APH 0-30	0,0003	S16b	60 vs. 90	0,0242
	Control 2h vs. APH 30-60	0,0035		90 vs. 120	0,0008
	Control 2h vs. APH 60-90	0,002		90 vs. 150	0,0271
	APH 2h vs. APH 90-120	0,0331		120 vs. 210	0,0311
				120 vs. 240	0,0323

Supplementary Table S2. *P*-values from statistical analysis. *P*-values obtained after statistical analysis for each Figure indicated, when comparing the results of two different experimental groups. Only statistically significant values are shown.
